# 2024 National Clinical Database annual report by the Japan surgical society

**DOI:** 10.1007/s00595-026-03266-4

**Published:** 2026-04-07

**Authors:** Takehito Yamamoto, Arata Takahashi, Tomoharu Yoshizumi, Soichiro Ishihara, Masafumi Inomata, Shigeru Imoto, Hidetoshi Eguchi, Tomoki Ebata, Masayuki Otsuka, Hiroomi Okuyama, Yoshihiro Kakeji, Tatsuya Kato, Takashi Kamei, Yoshikatsu Saiki, Aya Saito, Hideyuki Shimizu, Yoshiharu Soga, Tatsuro Tajiri, Hiroko Nogi, Etsuro Hatano, Hisato Hara, Yuko Bitoh, Tsunekazu Mizushima, Kenji Minatoya, Shigeru Miyagawa, Hideko Yamauchi, Ichiro Yoshino, Hideo Baba, Hisahiro Matsubara, Takao Ohki, Kiyoshi Hasegawa, Akinobu Taketomi

**Affiliations:** 1 Japan Surgical Society, Tokyo, Japan; 2 Department of Surgery, Graduate School of Medicine, Kyoto University, Kyoto, Japan; 3 Department of Healthcare Quality Assessment, Graduate School of Medicine, The University of Tokyo, Tokyo, Japan; 4 Department of Surgery and Science, Graduate School of Medical Sciences, Kyushu University, 3-1-1, Maidashi, Higashi-Ku, Fukuoka, 812-8582 Japan; 5 Department of Surgical Oncology, Graduate School of Medicine, The University of Tokyo, Tokyo, Japan; 6 Department of Gastroenterological and Pediatric Surgery, Oita University Faculty of Medicine, Oita, Japan; 7 Department of Breast Surgery, Kyorin University School of Medicine, Tokyo, Japan; 8 Department of Gastroenterological Surgery, Osaka University Graduate School of Medicine, Osaka, Japan; 9 Division of Surgical Oncology, Nagoya University Graduate School of Medicine, Aichi, Japan; 10 Department of General Surgery, Graduate School of Medicine, Chiba University, Chiba, Japan; 11 Department of Pediatric Surgery, Osaka University Graduate School of Medicine, Osaka, Japan; 12 Division of Gastrointestinal Surgery, Department of Surgery, Kobe University Graduate School of Medicine, Hyogo, Japan; 13 Department of Thoracic Surgery, Hokkaido University Graduate School of Medicine, Hokkaido, Japan; 14 Department of Surgery, Tohoku University Graduate School of Medicine, Miyagi, Japan; 15 Division of Cardiovascular Surgery, Tohoku University Graduate School of Medicine, Miyagi, Japan; 16 Department of Surgery, Yokohama City University Graduate School of Medicine, Kanagawa, Japan; 17 Department of Cardiovascular Surgery, Keio University School of Medicine, Tokyo, Japan; 18 Department of Cardiovascular Surgery, Graduate School of Medical and Dental Sciences, Kagoshima University, Kagoshima, Japan; 19 Department of Pediatric Surgery, Graduate School of Medical Sciences, Kyushu University, Fukuoka, Japan; 20 Department of Breast and Endocrine Surgery, Jikei University School of Medicine, Tokyo, Japan; 21 Division of Hepato-Biliary-Pancreatic Surgery and Transplantation, Department of Surgery, Kyoto University Graduate School of Medicine, Kyoto, Japan; 22 Institute of Medicine, Breast and Endocrine Surgery, University of Tsukuba, Ibaraki, Japan; 23 Division of Pediatric Surgery, Department of Surgery, Kobe University Graduate School of Medicine, Hyogo, Japan; 24 Department of Colorectal Surgery, Dokkyo Medical University, Tochigi, Japan; 25 Department of Cardiovascular Surgery, Graduate School of Medicine, Kyoto University, Kyoto, Japan; 26 Department of Cardiovascular Surgery, Osaka University Graduate School of Medicine, Osaka, Japan; 27 University of Hawai’i Cancer Center, Honolulu, USA; 28 International University of Health and Welfare Narita Hospital, Chiba, Japan; 29 The Chemo-Sero-Therapeutic Research Institute, Kumamoto, Japan; 30 Department of Frontier Surgery, Graduate School of Medicine, Chiba University, Chiba, Japan; 31 Division of Vascular Surgery, Department of Surgery, The Jikei University School of Medicine, Tokyo, Japan; 32 Hepato-Biliary-Pancreatic Surgery Division, Department of Surgery, Graduate School of Medicine, The University of Tokyo, Tokyo, Japan; 33 Department of Gastroenterological Surgery I, Hokkaido University Graduate School of Medicine, Hokkaido, Japan

**Keywords:** Japan surgical society, JSS, National Clinical Database, NCD

The Japan Surgical Society (JSS) has issued annual reports on national surgical activity using data derived from the National Clinical Database (NCD). The NCD began its case registration in 2011 with 3,374 participating institutions and 4916 clinical departments, capturing approximately 1.17 million cases in the first year [1]. By 2025, participation had expanded substantially, reaching 5,802 institutions, and the number of cumulated cases has reached more than 20 million, reflecting the remarkable nationwide dissemination of this registry. The NCD now covers more than 95% of surgeries performed in Japan and serves as an essential platform for surgical quality assessment and several board certification systems.

Since 2011, annual summaries of procedure volumes based on the NCD have been made available in Japanese language on the JSS website [2]. In 2024, for the first time, it was published as a scientific article in *Surgery Today*, the official journal of the JSS [3]. The present article represents the second such report and summarizes the findings for the 2024 dataset. In addition to presenting the most recent annual statistics, this report newly provides four-year temporal trends from 2021 to 2024. These longitudinal data offer an important resource for understanding current patterns of surgical practice in Japan and are expected to support a variety of future research.

The data used for this report were extracted within a secure, closed system. When interpreting the number of surgeries, the following points should be noted: First, up to eight procedures can be recorded for a single case in the NCD; hence, the total numbers presented in this report represent the sum of all registered procedures, rather than the total number of surgical cases. Second, the registry contains several procedures performed in internal medicine, making it difficult to capture the total number of such procedures accurately. Third, some procedures may appear across multiple specialty categories. Fourth, procedures with fewer than 20 cases were omitted from publication. Fifth, the English terminology used in this report reflects translations of procedure names uniquely defined within the NCD. Many surgeries performed on the same anatomical region are subdivided into multiple procedure categories. Readers referring the tables to identify the volume of a specific procedure should consider that the target procedure may be listed as several subdivided names.

## Survey abstract

All data were obtained from the NCD. The surgical procedures included in the present report were classified into seven categories covered by JSS:

I. Gastroenterological and abdominal surgery
II. Breast surgery
III. General thoracic surgery
IV. Cardiovascular surgery
V. Peripheral vascular surgery
VI. Surgery of the head and neck/body surface/endocrine system
VII. Pediatric surgery.

Figure 1 shows the trends in surgical procedure volumes from 2021 to 2024 in each category. All tables in this report list surgical procedures arranged in descending order of the number of surgeries performed.

**Fig. 1:**
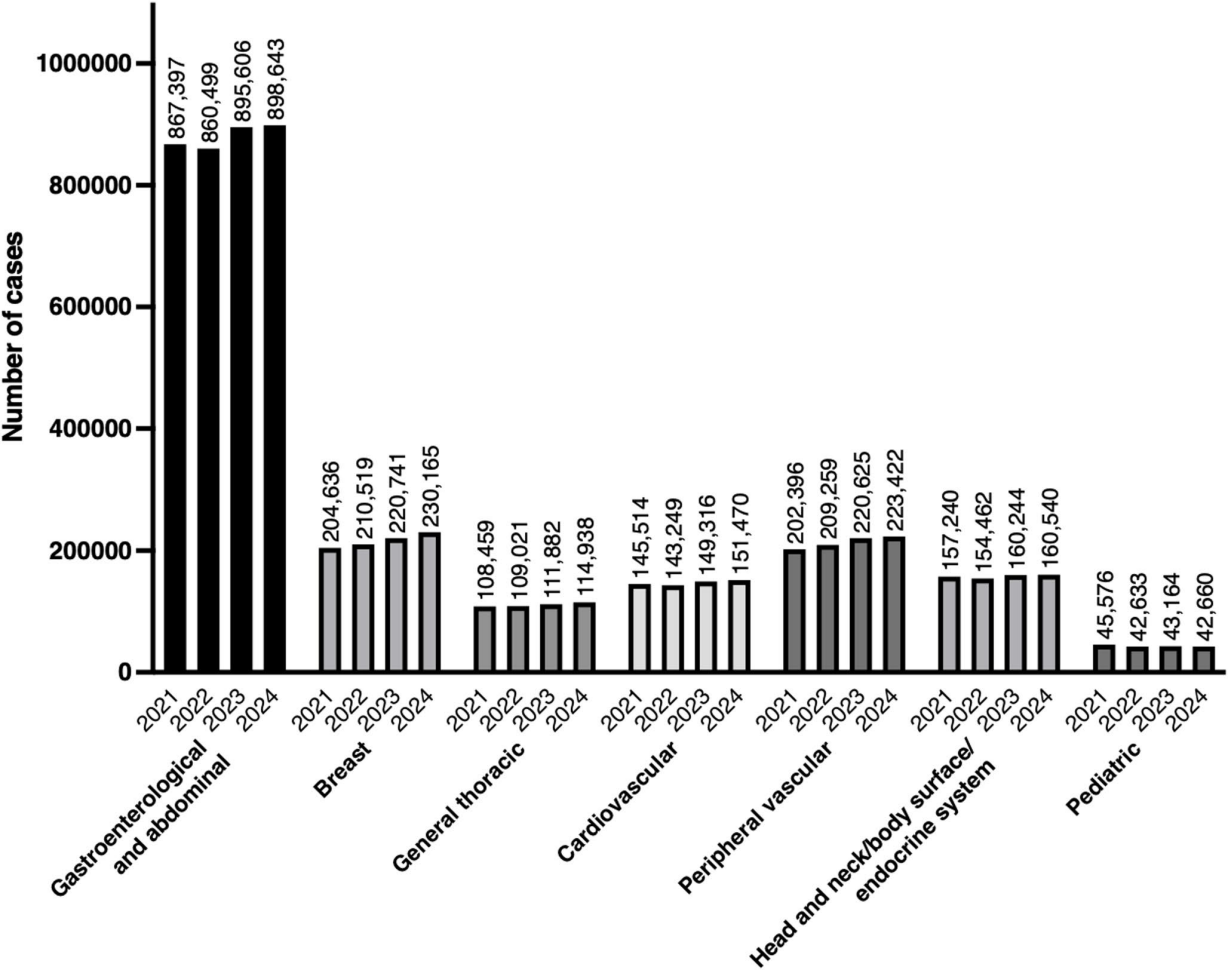
Trends in surgical procedure volumes from 2021 to 2024 across the seven categories defined by the Japan Surgical Society

### Gastroenterological and abdominal surgery

In 2024, there were 898,643 gastroenterological and abdominal surgical procedures registered in the NCD. This number remained largely unchanged compared with 2023, but shows a modest upward trend when viewed over the longer period preceding 2023. The procedure with the highest number in this category was laparoscopic cholecystectomy (n = 120,625), followed by laparoscopic inguinal hernia surgery (n = 87,819), and open inguinal hernia surgery (n = 59,483). Compared with the volumes in 2023, the number of laparoscopic inguinal hernia surgery increased by more than 5000 cases, whereas the volume of open inguinal hernia surgery decreased by approximately 3700 cases [3]. Tables 1, 2, 3, 4, 5, 6, 7, 8, 9 and Fig. 2 show the number of surgeries in each subcategory from 2021 to 2024 (esophagus; stomach and duodenum; intestine, appendix and colon; rectum and anus; liver, bile duct and pancreas; spleen; abdominal cavity and peritoneum; transplantation; and other gastroenterological and abdominal surgery, respectively). Procedures in the stomach/duodenum subcategory have shown a clear downward trend, with the most notable decline observed in gastrectomies registered as “extended resection for gastric malignancy.” In contrast, procedures in the rectum/anus category and the abdominal cavity/peritoneum subcategory have consistently increased. Compared with last year, the rise in laparoscopic inguinal hernia surgery contributed substantially to this increase in the abdominal cavity/peritoneum subcategory, and additional growth appears to be attributable to higher numbers of umbilical hernia surgery, abdominal wall incisional hernia surgery, exploratory laparoscopy for diagnosis, and Laparoscopic diffuse peritonitis surgery.

**Table 1:** Esophagus (total; 10,350 cases)

Procedure	Case
Thoracoscopic esophagectomy with gastrointestinal reconstruction (involving cervical, thoracic, and abdominal procedures, without vascular anastomosis) for a malignant tumor	4949
Esophageal hiatus hernia surgery (laparoscopic)	1224
Thoracoscopic esophagectomy with gastrointestinal reconstruction (involving thoracic and abdominal procedures, without vascular anastomosis) for a malignant tumor	542
Laparoscopic fundoplication	444
Thoracoscopic esophagectomy with gastrointestinal reconstruction (involving cervical, thoracic, and abdominal procedures, with vascular anastomosis) for a malignant tumor	293
Mediastinoscopic esophagectomy with gastrointestinal reconstruction (involving cervical, thoracic, and abdominal procedures, without vascular anastomosis) for a malignant tumor	267
Endoscopic esophagectomy for malignant tumor (resection only)	253
Esophageal hiatus hernia surgery (transabdominal)	233
Esophageal fistula construction	198
Laparoscopic fundoplication	160
Secondary esophageal reconstruction	158
Open esophagectomy with gastrointestinal reconstruction (involving thoracic and abdominal procedures) for a malignant tumor	119
Esophagectomy (resection only) of the thoracic esophagus	116
Secondary gastrointestinal reconstruction following esophagectomy (with vascular anastomosis)	115
Open esophagectomy with gastrointestinal reconstruction (involving cervical, thoracic, and abdominal procedures, without vascular anastomosis) for a malignant tumor	102
Esophagectomy with gastrointestinal reconstruction (involving abdominal procedures, including a transhiatal approach) for a malignant tumor	96
Esophageal reconstruction with bypass creation	86
Esophageal suture (perforation, injury) (thoracotomy)	81
Cervical esophagectomy with gastrointestinal reconstruction (involving cervical and abdominal procedures) for a malignant tumor	80
Esophageal perforation or injury repair (via laparotomy)	73
Mediastinoscopic esophagectomy with gastrointestinal reconstruction (involving thoracic and abdominal procedures, without vascular anastomosis) for a malignant tumor	68
Esophagectomy (resection only) of the abdominal esophagus	60
Mediastinoscopic esophageal resection with gastrointestinal reconstruction (without thoracotomy)	56
Achalasia surgery (laparoscopic)	54
Open fundoplication	53
Esophagectomy (resection only) of the thoracic esophagus	49
Esophageal resection and reconstruction for benign disease (involving abdominal procedures)	44
Esophageal suture (perforation, injury) (cervical approach)	39
Esophagectomy (resection only) of the cervical esophagus	39
Esophageal resection (resection only) of the abdominal esophagus	37
Thoracoscopic benign esophageal tumor removal	37
Gastric tube resection	36
Cervical esophagectomy with gastrointestinal reconstruction (involving cervical, thoracic, and abdominal procedures) for a malignant tumor	31
Surgery for a malignant tumor of the larynx and hypopharynx,with reconstruction involving cervical, thoracic, and abdominal procedures	26
Esophageal resection and reconstruction for benign disease (involving cervical, thoracic, and abdominal procedures)	24
Thoracotomy with drainage of peri-esophageal abscess	22
Esophageal foreign body extraction (cervical)	22
Thoracoscopic Esophageal diverticulum resection	22
Cervical incision and drainage of peri-esophageal abscess	21
Cervical Esophageal diverticulum resection	21
Total	10,350

**Table 2:** Stomach and duodenum (total; 57,723 cases)

Procedure	Case
Laparoscopic resection for gastric malignancy	17,556
Extended resection for gastric malignancy	6300
Laparoscopic total gastrectomy for gastric malignancy	3426
Gastrojejunostomy	3056
Gastrointestinal anastomosis	2678
Extended total gastrectomy without pedicled bowel transplantation	2634
Laparoscopic local gastrectomy	2290
Omental filling or covering procedure for gastric or duodenal ulcer perforation	2199
Gastric fistula construction	1792
Proximal gastrectomy for malignancy	1738
Laparoscopic-assisted gastrostomy (including percutaneous endoscopic, percutaneous, and open approaches)	1588
Laparoscopic endoscopic cooperative local gastrectomy	1344
Omental filling or covering procedure	1299
Simple resection for gastric malignancy	1044
Duodenal suture (perforation, injury) (laparoscopic)	993
Gastric suture (perforation, injury) (laparotomy)	890
Laparoscopic sleeve gastrectomy	797
Extended proximal gastrectomy for gastric malignancy	746
Open local gastrectomy	713
Simple total gastrectomy for gastric malignancy	631
Completion total gastrectomy	626
Open duodenal repair (for perforation, rupture, or injury)	598
Gastric suture (perforation, injury) (laparoscopic)	568
Gastrectomy for non-malignant disease	497
Duodenojejunostomy	256
Surgery for hypertrophic pyloric stenosis	246
Extended total gastrectomy with pedicled bowel transplantation	224
Open gastrostomy closure	147
Laparoscopic sleeve gastrectomy with bypass	99
Pylorus-preserving gastrectomy	95
Laparoscopic extended total gastrectomy with a jejunal pouch	88
Gastric foreign body extraction	85
Completion gastrectomy	82
Open pyloroplasty	71
Total gastrectomy for benign disease	51
Open duodenal polypectomy	47
Laparoscopic surgery for hypertrophic pyloric stenosis	46
Laparoscopic surgery for gastric volvulus	43
Surgery for gastric volvulus	35
Proximal gastrectomy for benign disease	30
Duodenal diverticulum resection	29
Laparoscopic pyloroplasty	23
Gastropexy for gastroptosis	23
Total	57,723

**Table 3:** Intestine, appendix, and colon (total; 254,259 cases)

Procedure	Case
Laparoscopic colectomy for a malignant tumor	50,756
Laparoscopic simple appendectomy	38,838
Stoma creation	33,206
Open small bowel resection	15,516
Laparoscopic complex appendectomy	15,427
Extended colectomy for a malignant tumor	14,780
Open surgery for intestinal adhesions	12,724
Stoma closure with enterectomy	11,869
Open partial colectomy	8799
Laparoscopic surgery for intestinal adhesions	8294
Laparoscopic partial colectomy	6628
Laparoscopic small bowel resection	5202
Open appendectomy	4215
Enterostomy creation	4159
Hemicolectomy	3365
Intestinal anastomosis	3289
Simple colectomy for malignant tumor	2897
Laparoscopic colectomy	2862
Stoma closure without enterectomy	2142
Stoma closure after Hartmann’s procedure	1337
Simple small bowel resection for malignant tumor	829
Subtotal colectomy	638
Laparoscopic small bowel resection for malignant tumor	632
Gastrointestinal perforation closure	445
Colostomy creation	435
Extended small bowel resection for malignant tumor	399
Enterostomy closure with enterectomy	398
Total colectomy	359
Meckel’s diverticulectomy	275
Enterotomy for foreign body extraction	271
Surgery for intestinal malrotation	270
Total colectomy with ileoanal anastomosis	261
Open stoma revision surgery	230
Small bowel seromuscular suture	220
Enterostomy closure without enterectomy	185
Laparoscopic total colectomy	182
Non-open stoma revision	174
Disinvagination (invasive) (including Hutchinson’s maneuver)	173
Laparoscopic enterostomy and appendicostomy creation	162
Small bowel tumor resection	160
Simple colonic suture	121
Small bowel diverticulectomy	109
Colostomy closure with colectomy	100
Total proctocolectomy with ileal pouch-anal anastomosis	94
Colonic seromuscular suture	90
Surgery for congenital intestinal atresia with bowel resection	84
Enterotomy and suture repair for intestinal stricture	83
Laparoscopic disinvagination (including Hutchinson’s maneuver)	77
Simple mesenteric injury surgery with enterectomy	73
Laparoscopic surgery for intestinal malrotation	66
Complex mesenteric injury surgery with enterectomy	59
Colostomy closure without colectomy	57
Surgery for congenital intestinal atresia without bowel resection	48
Open colonic polypectomy	41
Enterotomy for biopsy	38
Laparoscopic Hirschsprung disease surgery	36
Surgery for intestinal duplication	29
Colon tumor resection	26
Appendectomy and cecopexy	25
Total	254,259

**Table 4:** Rectum and anus (total; 158,221 cases)

Procedure	Case
Radical hemorrhoid surgery	39,102
Internal hemorrhoid surgery (four-step injection)	18,099
Radical surgery for complex fistula-in-ano	12,407
Laparoscopic low anterior resection	9993
Incision and drainage of perianal abscess	8978
Radical surgery for simple fistula-in-ano	8659
Low rectal surgery for malignant tumor (extensive resection)	7758
Laparoscopic high anterior resection	6328
High rectal surgery for malignant tumor (extensive resection)	5157
Laparoscopic abdominoperineal resection	3860
Anal polyp resection	3688
Hemorrhoid surgery (ligation)	3592
Radical surgery for anal fissure or ulcer	3021
Rectal prolapse surgery (transanal approach)	2813
Rectal resection	2533
Hemorrhoid surgery (sclerotherapy)	2381
Laparoscopic rectal prolapse surgery	2087
Anoplasty (rectal mucosal prolapse repair)	1989
Hemorrhoid surgery (thrombectomy)	1893
Anoplasty (for anal stricture)	1605
Rectal surgery for malignant tumor (extended amputation)	1311
Ultra-low anterior resection with transanal coloanal pouch anastomosis	1290
Anal dilation with internal sphincterotomy (invasive)	1287
Incision and drainage of perirectal abscess	1251
Transanal rectal tumor resection (including polyps)	1163
Hemorrhoid surgery (PPH)	693
Anal condyloma acuminatum removal	664
Open abdominoperineal resection	622
Abdominoperineal rectal prolapse surgery (including bowel resection)	505
Rectal surgery for malignant tumor (simple resection)	447
Total pelvic exenteration	417
Benign anal tumor resection	416
Rectal prolapse surgery (rectopexy)	393
Pilonidal cyst or sinus surgery	345
Transanal anal stricture dilation	222
Anal sphincteroplasty (tissue replacement)	154
Full-thickness rectal biopsy	153
Anal surgery for malignant tumor (simple resection)	145
Transanal intraluminal rectal tumor resection (including polyps)	140
Anal sphincteroplasty (scar excision or repair)	134
Hemorrhoid surgery (cauterization)	115
Partial rectal resection	107
Simple rectal suture	91
Open rectal foreign body extraction	45
Rectal stricture repair	45
Rectal seromuscular suture	41
Anal surgery for malignant tumor (extended resection)	34
Transsphincteric rectal tumor resection (including polyps)	25
Rectal surgery for malignant tumor (extensive resection with sacral resection)	23
Total	158,221

*PPH* Procedure for prolapse and hemorrhoids

**Table 5:** Liver, bile duct and pancreas (total; 185,673 cases)

Procedure	Case
Laparoscopic cholecystectomy	120,625
Open cholecystectomy	13,350
Pancreaticoduodenectomy with lymphadenectomy and nerve plexus dissection	8456
Laparoscopic partial hepatectomy (others)	6219
Partial hepatectomy (others)	4586
Hepatectomy (bi-sectionectomy)	2277
Hepatectomy (one-sectionectomy, excluding left lateral sectionectomy)	1657
Laparoscopic distal pancreatectomy with splenectomy and lymphadenectomy, with postoperative pathology confirming a primary invasive pancreatic malignancy or neuroendocrine tumor	1492
Pancreaticoduodenectomy with arterial or portal vein reconstruction	1481
Hepatic segmentectomy	1429
Laparoscopic hepatic segmentectomy	1333
Distal pancreatectomy with splenectomy and lymphadenectomy, with postoperative pathology confirming a primary invasive pancreatic malignancy or neuroendocrine tumor	1230
Laparoscopic distal pancreatectomy with splenectomy (others)	1198
Laparoscopic hepatic cyst fenestration	1130
Distal pancreatectomy with splenectomy (others)	941
Pancreaticoduodenectomy	929
Choledochotomy with stone extraction	900
Laparoscopic pancreaticoduodenectomy	823
Laparoscopic hepatectomy (bi-sectionectomy)	708
Laparoscopic hepatectomy (one-sectionectomy, excluding left lateral sectionectomy)	700
Choledochoenterostomy	698
cholecystectomy combined with gallbllader bed resction and lymphandenectomy for malignant gallbladder tumors (≥ pT2)	641
Surgery for localized malignant gallbladder tumor	601
Laparoscopic hepatic left lateral sectionectomy	584
Laparoscopic choledochotomy with stone extraction	583
cholecystectomy combined with gallbladder bed resction (others)	568
Hepatic left lateral sectionectomy	558
Partial hepatectomy (resection depth ≥ 5 cm)	545
partial hepatectomies (≥ 3 sites, at least one resection depth ≥ 3 cm)	494
Total pancreatectomy without vascular reconstruction	474
Distal pancreatectomy with lymphadenectomy and nerve plexus dissection with postoperative pathology confirming primary invasive pancreatic malignancy or neuroendocrine tumor	466
Resection of bile duct malignancy with hepatectomy	435
Laparoscopic partial hepatectomy with main tumor location in S4a, S7, or S8, with tumor ≥ 4 cm or resection depth ≥ 4 cm)	432
Liver biopsy (needle puncture)	427
Pancreaticoduodenectomy with resection of adjacent organs	323
Hilar cholangiocarcinoma resection without vascular reconstruction	309
Vascular (or venous) grafting or bypass	308
Distal pancreatectomy with lymphadenectomy and nerve plexus dissection (others)	292
Distal pancreatectomy with resection of adjacent organs and lymphadenectomy with postoperative pathology confirming primary invasive pancreatic malignancy or neuroendocrine tumor	287
Gallbladder malignancy resection with hepatectomy (segment 4a + 5 or more)	254
Laparoscopic spleen-preserving distal pancreatectomy (others)	250
Bile duct malignancy resection with lymphadenectomy	246
Bile duct resection	210
Extrahepatic bile duct resection with cholecystectomy and bile duct reconstruction	205
Central pancreatectomy	204
Pancreaticoduodenectomy with artery and portal vein reconstruction	188
Pancreaticojejunostomy (others)	177
External cholecystostomy	176
Hepatectomy (trisegmentectomy)	161
Distal pancreatectomy with resection of adjacent organs (others)	158
Laparoscopic distal pancreatectomy with splenic vessel preservation	155
Hepatorrhaphy	147
Pancreatic tumor resection	143
Laparoscopic spleen-preserving distal pancreatectomy with lymphadenectomy and postoperative pathology confirming primary invasive pancreatic malignancy or neuroendocrine tumor	111
Resection of hilar cholangiocarcinoma with vascular reconstruction	110
Spleen-preserving distal pancreatectomy (others)	110
Malignant bile duct tumor surgery with hepatectomy and pancreaticoduodenectomy	108
External biliary fistula	107
Bilioenteric anastomosis	103
Partial hepatectomy including the paracaval portion of the caudate lobe	98
Distal pancreatectomy with vascular reconstruction accompanied by lymphadenectomy, with postoperative pathology confirming a primary invasive pancreatic malignancy or neuroendocrine tumor	95
Laparoscopic surgery for choledochal cyst	91
Laparoscopic partial hepatectomy for a tumor located primarily in the caudate lobe with dissection from the inferior vena cava with short hepatic vein resection	91
Open hepatic cyst fenestration	87
Caudate lobectomy	86
Esophageal varices ligation	85
Robot-assisted surgery for choledochal cysts	82
Surgery for congenital biliary atresia	82
Cysticolithectomy	79
Distal pancreatectomy with splenic artery and vein preservation	67
Laparoscopic transcholecystic extraction of bile duct stones	65
Biliary tract reconstruction	63
Total pancreatectomy with arterial or portal vein reconstruction	58
Pancreaticojejunostomy (Frey procedure)	56
Total pancreatectomy with simultaneous arterial and portal vein reconstruction	55
Malignant gallbladder tumor surgery with pancreaticoduodenectomy	55
Ampullectomy	51
Percutaneous drainage of liver abscess	47
Hepatectomy with vascular reconstruction	46
Spleen-preserving distal pancreatectomy accompanied by lymphadenectomy, with postoperative pathology confirming a primary invasive pancreatic malignancy or neuroendocrine tumor	46
Acute pancreatitis surgery	40
Hepatic injury ablation and coagulation hemostasis (including hemostatic agents)	39
Open transcystic choledocholithotomy	37
Deceased donor pancreas procurement	36
Jejunal interposition of bile duct	33
Choledochotomy	32
Necrosectomy of pancreas	31
Hepatic cyst resection	29
Distal pancreatectomy with vascular reconstruction (others)	28
Duodenum-preserving pancreatic head resection	25
Drainage of peripancreatic injury site	24
Cholecystoenterostomy	22
Total	185,673

**Table 6:** Spleen (total; 1578 cases)

Procedure	Case
Open splenectomy	925
Laparoscopic splenectomy	617
Partial splenectomy	36
Total	1578

**Table 7:** Abdominal cavity and peritoneum (total; 216,744 cases)

Procedure	Case
Laparoscopic inguinal hernia surgery	87,819
Open inguinal hernia surgery	59,483
Acute diffuse peritonitis surgery with drainage of intraperitoneal abscess	13,494
Umbilical hernia surgery (diastasis recti)	9719
Exploratory laparoscopy for diagnosis	7419
Exploratory laparotomy for diagnosis and biopsy	6576
Open abdominal wall incisional hernia surgery	5627
Laparoscopic abdominal wall incisional hernia surgery	4906
Laparoscopic diffuse peritonitis surgery	4082
Open femoral hernia surgery	2980
Laparoscopic femoral hernia surgery	2083
Obturator hernia surgery	2075
Omental, mesenteric, and retroperitoneal tumor resection (without bowel resection)	1514
Laparoscopic tumor biopsy	1230
Localized intraperitoneal abscess surgery (other types)	880
Localized intraperitoneal abscess surgery (appendiceal abscess)	878
Temporary vacuum packing abdominal closure	689
Internal hernia surgery	670
Omentectomy	572
Linea alba hernia surgery	489
Simple resection of retroperitoneal malignant tumor	410
Omental, mesenteric, and retroperitoneal tumor resection (with bowel resection)	384
Laparoscopic resection of urachus (cyst)	324
Laparoscopic retroperitoneal tumor resection	276
Extended resection of retroperitoneal malignant tumor	255
Surgery for mesenteric injury (suture repair only)	210
Localized intraperitoneal abscess surgery (Douglas pouch abscess)	144
Diaphragm suture repair (transabdominal, without patch reconstruction)	126
Surgery for abdominal wall fistula (communicating with the abdominal cavity)	116
Thoracoscopic diaphragm plication	112
Lumbar hernia surgery	110
Multivisceral resection with peritonectomy	101
Semilunar line hernia surgery	93
Thoracoscopic diaphragm suture repair (without patch reconstruction)	92
Localized intraperitoneal abscess surgery (subphrenic abscess)	90
Decompressive laparotomy for abdominal compartment syndrome	87
Diaphragm suture repair (transthoracic, without patch reconstruction)	70
Diaphragmatic hernia surgery	61
Laparoscopic diaphragm suture repair (without patch reconstruction)	61
Retroperitoneal abscess drainage	57
Transabdominal diaphragm plication	54
Removal of perihepatic packing gauze	53
Abdominal wall reconstruction (including skin grafting)	52
Perineal hernia surgery	51
Component separation technique	48
Transthoracic diaphragm plication	47
Combined thoracoabdominal diaphragm suture repair (without patch reconstruction)	30
Combined thoracoabdominal diaphragm suture repair (with patch reconstruction)	25
Laparoscopic diaphragm plication	20
Total	216,744

**Table 8:** Transplantation (total; 2593 cases)

Procedure	Case
Renal transplantation	784
Laparoscopic donor nephrectomy	547
Living donor liver transplantation	358
Back table preparation for living donor liver transplantation	186
Living donor right hemi-hepatectomy	146
Deceased donor liver transplantation	117
Liver procurement from deceased donor	101
Living donor left hemi-hepatectomy	87
Living donor left lateral sectionectomy	80
Back table preparation for deceased donor liver transplantation	56
Kidney procurement from deceased donor	50
Deceased donor pancreas transplantation	44
Living donor left hemi-hepatectomy with segmentectomy 1 (caudate segmentectomy)	37
Total	2593

**Table 9:** Other gastroenterological and abdominal surgery (total; 11,502 cases)

Procedure	Case
Hartmann’s procedure	6969
Open hemostasis surgery	1239
Abdominal paracentesis with filtration, concentration, and reinfusion	717
Percutaneous RFA for a malignant liver tumor	377
Damage control surgery for hemorrhagic shock in abdominal trauma	302
Stent placement in the small intestine, colon, or rectum	222
Disinvagination (invasive) (including high-pressure enema)	219
Placement of an implantable catheter for continuous intraperitoneal infusion	177
Thoracotomy for hemostasis	173
Open RFA for a malignant liver tumor	129
Peroral endoscopic myotomy	129
Liver hemostasis (gauze packing)	117
Thoracentesis with filtration, concentration, and reinfusion	112
Percutaneous MWA for a malignant liver tumor	95
Sacral nerve stimulator lead insertion	68
Laparoscopic RFA for a malignant liver tumor	68
Open MWA for a malignant liver tumor	63
Pylorus-preserving pancreaticoduodenectomy	55
Laparoscopic high anorectal reconstruction for imperforate anus	52
Umbilical fistula surgery without bowel resection	41
Gastric vessel ligation for acute gastric bleeding	34
Mechanical dilation for upper gastrointestinal anastomotic stricture	34
Esophageal banding	32
Esophageal dilation with Tucker bougie	32
Esophageal foreign body extraction with a balloon	23
Gastric foreign body extraction with magnetic catheter	23
Total	11,502

*RFA* Radiofrequency ablation, *MWA* Microwave ablation

**Fig. 2:**
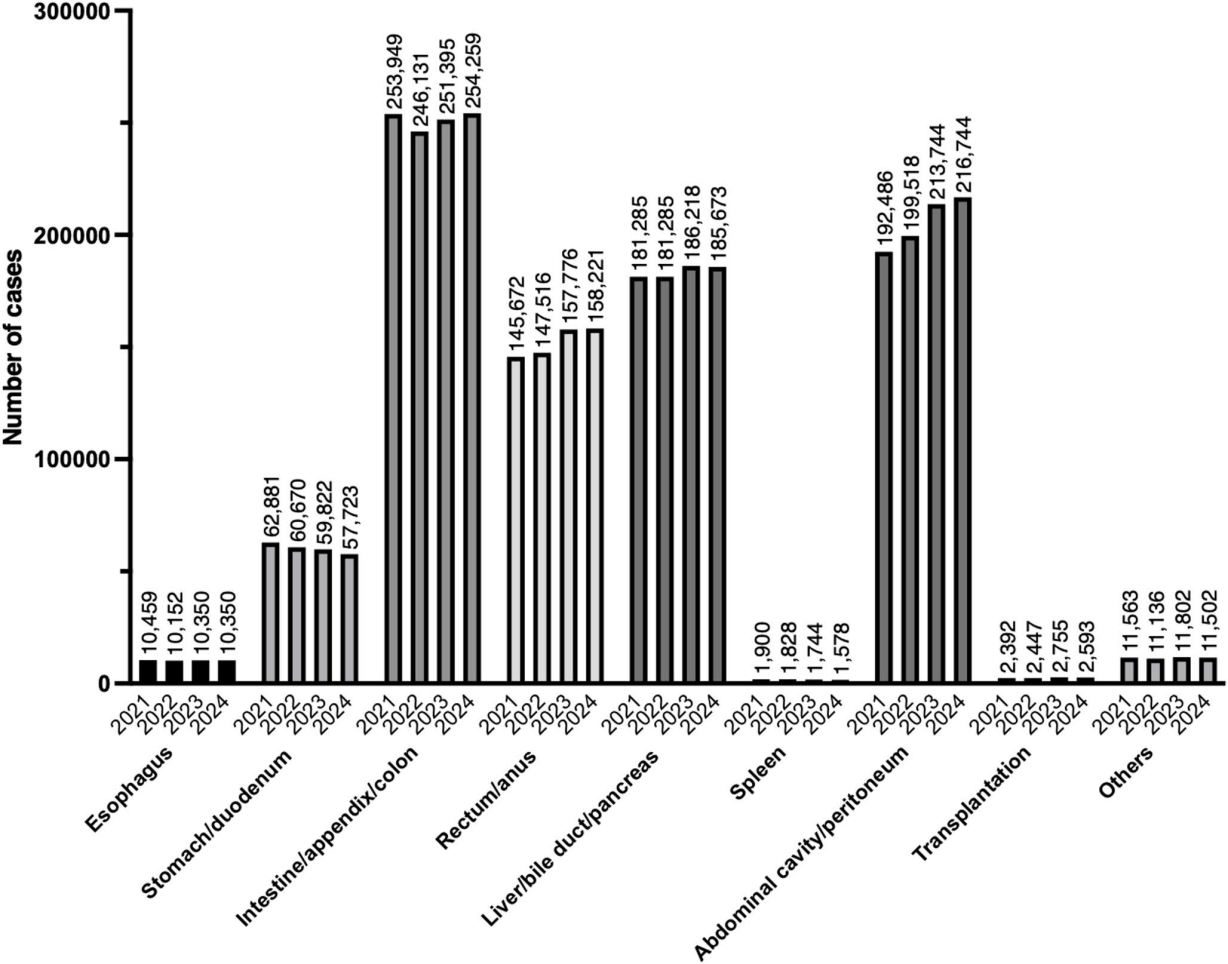
Trends in surgical procedure volumes from 2021 to 2024 in the category of gastroenterological and abdominal surgery

### Breast surgery

In 2024, there were 230,165 breast surgery procedures registered in the NCD. Since 2021, the number of registered cases in this category has increased by approximately 10,000 per year. Table 10 presents all the procedures in this category by volume. It should be noted that the total number of procedures does not correspond to the total number of cases, because multiple procedures, such as breast surgery and axillary surgery, may be registered for a single case.

**Table 10:** Breast surgery (total; 230,165 cases)

Procedure	Case
Sentinel lymph node biopsy for breast malignancy	64,313
Breast malignancy surgery: Mastectomy without axillary lymph node dissection	42,198
Breast malignancy surgery: Partial mastectomy without axillary lymph node dissection	39,411
Breast malignancy surgery: Mastectomy with axillary and infraclavicular lymph node dissection (without pectoral muscle resection)	18,767
Breast tissue biopsy by core needle biopsy	17,425
Image-guided vacuum-assisted breast biopsy	12,837
Breast tumor excision: Lesion < 5 cm (benign breast lesion)	5551
Lymph node excision: Lesion < 3 cm	5434
Breast malignancy surgery: Partial mastectomy with axillary lymph node dissection	5200
Axillary lymph node dissection	3094
Lymph node excision: Lesion ≥ 3 cm	2781
Breast tumor excision: Lesion ≥ 5 cm (benign breast lesion)	2661
Nipple-sparing mastectomy	1845
Immediate breast reconstruction after mastectomy (using implant)	1696
Skin-sparing mastectomy	1384
Breast malignancy surgery: Mastectomy with axillary and infraclavicular lymph node dissection (with pectoral muscle resection)	843
Mastectomy for benign breast lesion	786
Immediate breast reconstruction after mastectomy (using autologous tissue)	717
Breast abscess drainage	654
Delayed breast reconstruction (using implant)	547
Ductal lobular segmentectomy	496
Lymph node biopsy by core needle biopsy	389
Breast tissue biopsy by incisional biopsy (Incisional biopsy of breast tissue)	353
Breast malignancy surgery (RFA)	296
Supraclavicular and infraclavicular lymph node dissection	137
Excision of breast foreign body	80
Nipple reconstruction (for inverted nipple)	62
Nipple reconstruction (for reconstructed breast)	61
Radical surgery for subareolar abscess	54
Delayed breast reconstruction (using autologous tissue)	34
Breast malignancy surgery: Extended mastectomy with internal mammary, supraclavicular, and infraclavicular lymph node dissection	33
Parasternal lymph node dissection	26
Total	230,165

The procedure with the highest number in this category was sentinel lymph node biopsy for breast malignancy (n = 64,313), followed by mastectomy without axillary lymph node dissection (n = 42,198) and partial mastectomy without axillary lymph node dissection (n = 39,411), for breast malignancy. All three of these procedures increased substantially compared with the previous year, and a notable rise was also observed in the number of image-guided vacuum-assisted breast biopsies registered in the NCD [3].

### General thoracic surgery

In 2024, 114,938 general thoracic surgical procedures were registered in the NCD. Since 2021, the number of registered cases in this category has slightly and consistently increased every year. The procedure with the highest number in this category was thoracoscopic lobectomy with lymph node dissection for a malignant lung tumor (n = 25,233), followed by thoracoscopic wedge resection for a malignant lung tumor (single site) (n = 14,900), and thoracoscopic bullectomy (single site) (n = 10,776). Tables 11, 12, 13, 14, 15 and Fig. 3 show the number of surgeries in each subcategory (trachea, bronchi, and lung; chest wall, pleura, and diaphragm; mediastinum; transplantation; and other respiratory surgery, respectively).

**Table 11:** Trachea, bronchi, and lung (total; 86,425 cases)

Procedure	Case
Thoracoscopic lobectomy with lymph node dissection for a malignant lung tumor	25,233
Thoracoscopic wedge resection for a malignant lung tumor (single site)	14,900
Thoracoscopic bullectomy (single site)	10,776
Thoracoscopic segmentectomy with lymph node dissection for a malignant lung tumor	9656
Thoracoscopic wedge resection (single site)	4497
Lobectomy with lymph node dissection for a malignant lung tumor (thoracotomy)	3298
Thoracoscopic segmentectomy without lymph node dissection for a malignant lung tumor	3117
Thoracoscopic bullectomy (two or more sites)	1891
Thoracoscopic pulmorrhaphy	1791
Thoracoscopic wedge resection for a malignant lung tumor (two or more sites)	1600
Thoracoscopic lobectomy without lymph node dissection for a malignant lung tumor	1018
Thoracoscopic wedge resection (two or more sites)	935
Wedge resection for a malignant lung tumor	932
Thoracoscopic segmentectomy	900
Thoracoscopic lobectomy	879
Segmentectomy with lymph node dissection for a malignant lung tumor	647
Thoracoscopic lung biopsy	604
Wedge resection	558
Lobectomy	411
Segmentectomy without lymph node dissection for a malignant lung tumor	356
Pulmonary plication (thoracotomy)	285
Lung resection with bronchoplasty for a malignant lung tumor	283
Lung resection with chest wall for malignant lung tumor	233
Closure of bronchopleural fistula	215
Thoracoscopic lung resection (more than one lobe)	210
Lobectomy without lymph node dissection for a malignant lung tumor (thoracotomy)	205
Wedge resection with lymph node dissection for a malignant lung tumor	127
Segmentectomy	127
Pneumonectomy with lymph node dissection for a malignant lung tumor (thoracotomy)	104
Thoracoscopic lung resection with combined resection of other organs for a malignant lung tumor	91
Lung resection with combined resection of other organs for a malignant lung tumor	86
Thoracoscopic lung resection with bronchoplasty for a malignant lung tumor	66
Thoracoscopic lung resection with chest wall resection for a malignant lung tumor	52
Lung resection (combined resection of more than one lobe)	50
Pneumonectomy (complete removal of one lung)	34
Lung resection with pericardial resection for a malignant lung tumor	31
Lung resection for extralobar pulmonary sequestration	31
Thoracoscopic pneumonectomy with lymph node dissection for a malignant lung tumor	30
Lung resection with diaphragmatic resection for a malignant lung tumor	29
Lung resection with combined resection of the chest wall, pericardium, or diaphragm for a malignant lung tumor	26
Lung abscess drainage	26
Tracheoplasty (including segmental tracheal reconstruction/tracheal trasnplantation) via thoracotomy or median sternotomy	23
Thoracoscopic lobectomy with diaphragmatic resection for a malignant lung tumor	22
Lung resection with pulmonary vascular reconstruction	20
Closure of tracheoesophageal fistula	20
Total	86,425

**Table 12:** Chest wall, pleura, and diaphragm (total; 9,335 cases)

Procedure	Case
Thoracoscopic debridement for empyema	3563
Thoracoscopic decortication of empyema and pleural thickening	1107
Evacuation of intrathoracic (intrapleural) hematoma	738
Evacuation of intrathoracic hematoma	463
Open window thoracostomy	329
Thoracoscopic extirpation of a benign chest wall tumor	284
Chest wall or sternum tumor resection for a malignant tumor (without chest wall reconstruction)	253
Decortication of empyema exceeding the range of one lobe	226
Partial pleurectomy	212
Pectus excavatum repair by sternal elevation	189
Thoracoscopic resection of malignant pleural tumor	172
Space-filling of omentum flaps for an empyema cavity	140
Thoracoplasty for empyema (with rib resection)	139
Diaphragm suture repair (transabdominal, without patch reconstruction)	126
Decortication of empyema within the area of one pulmonary lobe	121
Thoracoscopic diaphragm plication	112
Thoracoscopic excision of a benign pleural tumor	111
Space-filling of pedicled muscle flaps for an empyema cavity	98
Pleurectomy/Pulmonary decortication	91
Chest wall/sternum resection for a malignant tumor (with chest wall reconstruction)	89
Thoracoscopic thoracic duct ligation for chylothorax	77
Resection of a malignant pleural tumor	72
Diaphragm suture repair (transthoracic, without patch reconstruction)	70
Excision of a benign chest wall tumor (with thoracotomy)	62
Congenital diaphragmatic hernia repair (transabdominal, direct suture)	60
Excision of a benign chest wall tumor (without thoracotomy)	55
Diaphragm plication (transthoracic)	47
Thoracoscopic surgery for congenital diaphragmatic hernia (direct suture)	46
Congenital diaphragmatic hernia repair (transabdominal, with synthetic patch)	44
Sternum resection	41
Chest wall resection for a malignant tumor (chest wall reconstruction with vascular anastomosis)	37
Pulmonary decortication exceeding the area of one pulmonary lobe	37
Extended pleurectomy/decortication	29
Thoracoplasty for empyema (with pleural decortication)	28
Excision of a benign pleural tumor	26
Pulmonary decortication within the area of one pulmonary lobe	21
Sternal osteomyelitis surgery or sternal caries surgery	20
Total	9335

**Table 13:** Mediastinum (total; 7209 cases)

Procedure	Case
Thoracoscopic resection of a mediastinal tumor	3313
Thoracoscopic resection of a malignant mediastinal tumor	1301
Thoracoscopic thymectomy	441
Thoracotomy resection of a mediastinal tumor	402
Malignant mediastinal tumor resection	268
Thoracoscopic extended thymectomy for myasthenia gravis	214
Malignant mediastinal tumor resection with combined resection of other organs	172
Thymectomy (thoracotomy)	171
Malignant mediastinal tumor resection with lung resection	151
Mediastinal abscess surgery	137
Malignant mediastinal tumor resection with pericardial resection	131
Mediastinoscopic Mediastinal biopsy	112
Thoracoscopic mediastinotomy	111
Extended thymectomy for myasthenia gravis (thoracotomy)	72
Non-thoracotomy resection of mediastinal tumor	68
Trans-thoracic mediastinotomy	42
Mediastinoscopic resection of mediastinal tumor	38
Malignant mediastinal tumor resection with superior vena cava resection	33
Cervical mediastinotomy	32
Total	7209

**Table 14:** Transplantation (total; 273 cases)

Procedure	Case
Procurement of bilateral lungs from a deceased donor for transplantation	69
Single lung transplantation from a deceased donor	65
Bilateral lung transplantation from a deceased donor	64
Procurement of a single lung from a deceased donor for transplantation	41
Procurement of a single lung from a living donor for transplantation	34
Total	273

**Table 15:** Other respiratory surgeries (total; 11,696 cases)

Procedure	Case
Exploratory thoracoscopy	2774
Thoracic drainage with continuous suction	1840
Thoracoscopic thoracic sympathectomy	1469
Exploratory thoracotomy	1376
Pericardiotomy	1328
Thoracoscopic mediastinal biopsy	573
Open reduction and fixation for rib fracture	466
Thoracoscopic pectus excavatum repair	305
Evacuation of mediastinal hematoma	239
Thoraco-peritoneal shunt valve placement	213
Thoracoscopic pericardiotomy	183
Rib resection	119
Thoracoscopic pleural adhesional ablation	99
Tracheostomy closure	95
Mediastinal lymphadenectomy	94
Tracheal stricture dilation	82
Chest wall abscess incision and drainage	72
Open reduction and fixation for sternum fracture	70
Extracorporeal membrane oxygenation (ECMO) initiation	59
Surgery for tracheal stenosis	41
Extracorporeal membrane oxygenation (ECMO) removal	36
Intrathoracic foreign body removal	31
Non-thoracotomy mediastinal biopsy	30
Lung biopsy by needle puncture	28
Open mediastinal biopsy	26
Thoracoscopic intrathoracic foreign body removal	24
Tracheal foreign body removal under fiberscope guidance	24
Total	11,696

*ECMO* Extracorporeal membrane oxygenation

**Fig. 3:**
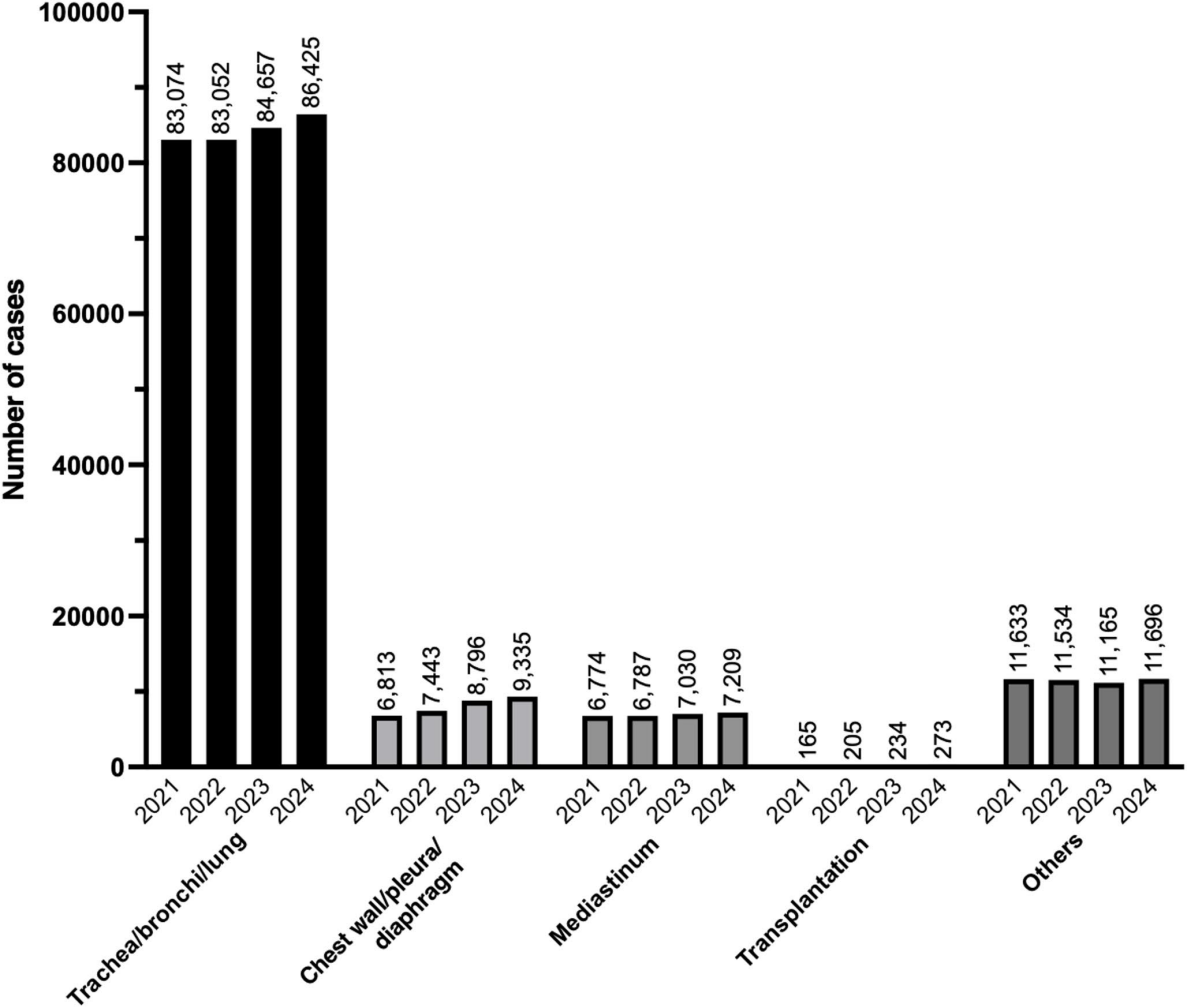
Trends in surgical procedure volumes from 2021 to 2024 in the category of general thoracic surgery

When examining the trend in recent 4 years, the trachea/bronchi/lung, chest wall/pleura, and mediastinum categories demonstrated upward trends. The largest year-to-year increase was observed in thoracoscopic segmentectomy with lymph node dissection for malignant lung tumors, which rose by approximately 1500 registered cases compared with last year. When combined with thoracoscopic segmentectomy without lymph node dissection for malignant lung tumors, the increase reached nearly 2000 cases [3]. In addition, thoracoscopic debridement for empyema also showed a consistent upward trend.

### Cardiovascular surgery

In 2024, 151,470 cardiovascular surgical procedures were registered in the NCD. Since 2022, the number of registered cases in this category has slightly and consistently increased every year. The procedure with the highest number in this category was stent grafting of the abdominal aorta (n = 13,206), followed by transcatheter aortic valve replacement (TAVR) without thoracotomy (n = 11,746), and aortic valve replacement (AVR) (n = 9,495).

Tables 16, 17, 18, 19, 20, 21, 22, 23, 24, 25 and Fig. 4 show the number of surgeries in each subcategory (all heart diseases, congenital heart disease, valvular heart disease, ischemic heart disease, transplantation, other heart diseases, other cardiac surgeries, great vessels, great veins, and other great vessel surgeries, respectively). The number of transcatheter aortic valve replacement (TAVR) without thoracotomy showed the largest year-to-year increase among all procedures, rising by 1050 cases from 10,696 in 2023 [3]. Moreover, when examining trends since 2021, the volume has exhibited a steady annual increase of approximately 1000 cases.

**Table 16:** All heart diseases (total; 11,371 cases)

Procedure	Case
Thoracoscopic exploratory thoracotomy	2774
Median sternotomy	2552
Pacemaker implantation with transvenous leads	2221
Exploratory thoracotomy	1376
Pericardiotomy	1328
Pacemaker implantation with myocardial leads	283
Myocardial suture hemostasis for traumatic injury	216
Thoracoscopic pericardial fenestration	183
Pericardial closure	154
Pacemaker lead implantation	83
Biventricular pacemaker implantation	76
Bilateral anterolateral thoracotomy (clam shell thoracotomy)	74
Pacemaker lead replacement	51
Total	11,371

**Table 17:** Congenital heart disease (total; 8428 cases)

Procedure	Case
ASD closure (alone)	1882
VSD closure (alone)	1446
Pulmonary artery reconstruction (including pulmonary vein trunk and main pulmonary artery)	862
PDA closure (open surgery)	831
Pulmonary artery banding	520
Systemic-to-pulmonary artery shunt (Blalock-Taussig shunt)	468
Subvalvular aortic stenosis resection (including fibrous and muscular thickening)	273
Tetralogy of Fallot repair (with ventriculotomy)	223
Fontan procedure	220
Bilateral pulmonary artery banding	204
Aortic coarctation repair (alone)	193
TGA repair (Rastelli procedure)	130
DORV repair (alone)	127
PAPVR repair	115
AVC (AVSD) repair, intermediate (transitional)	111
TAPVR repair (supracardiac and infracardiac types)	108
TGA repair (Jatene arterial switch operation)	87
Pulmonary artery de-banding	80
Coronary arteriovenous fistula ligation (via thoracotomy)	74
Aortic coarctation repair (with VSD closure)	58
Double-chambered right ventricle repair	53
Partial AVSD repair (ASD patch closure only)	49
Arterial switch operation (with VSD closure)	43
Tricuspid atresia repair (Fontan procedure)	41
Interrupted aortic arch repair (alone)	35
Valsalva sinus aneurysm repair (alone)	35
TAPVR repair (cardiac type)	31
Supravalvular aortic stenosis repair	30
Anomalous origin of coronary artery repair	29
Aortopulmonary septal defect repair (alone)	25
Tricuspid valve surgery (for Ebstein anomaly)	23
Valsalva sinus aneurysm repair (with aortic insufficiency repair)	22
Total	8428

*ASD* Atrial septal defect, *VSD* Ventricular septal defect, *PDA* Patent ductus arteriosus, *AVC* Atrioventricular canal, *AVSD* Atrioventricular septal defect, *TAPVR* Total anomalous pulmonary venous return, *PAPVR* Partial anomalous pulmonary venous return, *DORV* Double outlet right ventricle, *TGA* Transposition of the great arteries

**Table 18:** Valvular heart disease (total; 41,135 cases)

Procedure	Case
Transcatheter aortic valve replacement (TAVR) without thoracotomy	11,746
Aortic valve replacement (AVR)	9495
Mitral valvuloplasty	5724
Mitral valve replacement (MVR)	2193
Tricuspid valvuloplasty	1948
Valvuloplasty (Mitral and tricuspid valves)	1382
Ascending aortic aneurysm repair with aortic root and valve replacement	1368
Mitral valve replacement (MVR) and tricuspid valvuloplasty	953
Ascending aortic and arch repair with aortic valve replacement or valvuloplasty	599
Aortic and mitral valve replacement	591
Mitral and tricuspid valvuloplasty	543
Valve-sparing aortic root replacement	436
Aortic valvuloplasty	408
Ascending aortic and arch repair with aortic root and valve replacement	402
Pulmonary valve replacement	375
Aortic valve replacement with annular enlargement	364
Ascending aortic aneurysm repair with aortic valve replacement or valvuloplasty	354
Redo valve replacement (single valve)	338
Aortic valve replacement (AVR) and mitral valvuloplasty	336
Aortic valve replacement and mitral and tricuspid valvuloplasty	279
Aortic and mitral valve replacement with tricuspid valvuloplasty	276
Aortic valve replacement and tricuspid valvuloplasty	273
Transcatheter aortic valve replacement (TAVR) with thoracotomy	235
Tricuspid valve replacement	142
Ascending aortic and arch repair with valve-sparing aortic root replacement	109
Mitral and tricuspid valve replacement	76
Percutaneous transluminal mitral valvuloplasty	42
Ross operation (aortic root replacement with pulmonary autograft)	40
Triple valve replacement	33
Aortic and mitral valvuloplasty	29
Redo valve replacement (other two valves)	23
Redo valve replacement (two valves)	23
Total	41,135

**Table 19:** Ischemic heart disease (total; 17,309 cases)

Procedure	Case
CABG for two or more vessels	7100
Off-pump CABG for two or more vessels	5742
CABG for one vessel	2486
Off-pump CABG for one vessel	831
Ventricular septal rupture closure (alone)	268
Left ventricular free wall rupture repair (alone)	184
Surgical left ventricular restoration (alone)	173
Left ventricular free wall rupture repair	91
Ventricular aneurysm reconstruction (including infarctectomy, alone)	78
Ventricular septal rupture repair (alone)	68
Coronary endarterectomy (1 site)	60
Surgical ventricular restoration with CABG for two or more vessels	54
Ventricular septal rupture closure with CABG for one vessel	43
Ventricular septal rupture closure with CABG for two or more vessels	33
Surgical left ventricular restoration with CABG for one vessel	31
Ventricular aneurysm reconstruction with CABG for two or more vessels	24
Ventricular septal rupture repair with CABG for one vessel	22
Intracardiac myxoma excision with CABG for two or more vessels	21
Total	17,309

*CABG* Coronary artery bypass grafting

**Table 20:** Transplantation (total; 167 cases)

Procedure	Case
Allogeneic heart transplantation	111
Heart procurement for transplantation	56
Total	167

**Table 21:** Other heart diseases (total; 4,484 cases)

Procedure	Case
Arrhythmia surgery (Maze procedure)	2448
Pulmonary vein isolation	1127
Intracardiac myxoma excision (alone)	569
Surgical treatment of constrictive pericarditis	166
Thrombectomy of pulmonary artery	103
Malignant pericardial tumor resection	71
Total	4484

**Table 22:** Other cardiac surgeries (total; 19,534)

Procedure	Case
Left atrial appendage closure	4454
Surgical removal of PCPS/ECMO	3134
Left atrial appendage excision	2620
Cardiopulmonary bypass setup	1370
Surgical insertion of PCPS/ECMO	991
Re-sternotomy for hemostasis	983
Open-chest cardiac massage	617
PCPS insertion	460
Right ventricular outflow tract reconstruction	376
Intracardiac foreign body removal	299
Left atrial plication (LAP)	292
Single-ventricle repair (bidirectional Glenn procedure)	246
Mediastinal hematoma removal	239
Temporary epicardial pacing	238
ICD implantation	213
Delayed sternal closure	207
Exploratory open-heart surgery	193
Intra-atrial thrombus removal	191
ICD replacement	171
Implantation of an implantable VAD	163
Pulmonary artery debanding	162
VAD placement	144
Open atrial septostomy	131
Percutaneous transluminal septal myocardial ablation	105
Coronary artery aneurysm surgery	103
Transvenous lead extraction using laser sheath	103
Right ventricle to pulmonary artery conduit placement	99
Percutaneous transluminal septal myocardial ablation (with additional procedures)	92
ICD (with biventricular pacing function) implantation	89
VAD explantation	86
Implantable loop recorder placement	80
Retrograde coronary perfusion	75
Replacement of ICD with biventricular pacing function	71
Conduit reoperation	67
Biventricular pacemaker replacement	65
Norwood procedure for hypoplastic left heart syndrome	60
ECMO initiation	59
Vascular ring surgery	54
Cardiac reoperation (excluding staged procedures)	42
Removal of implantable cardiac rhythm management device	42
Shunt ligation and takedown	39
ECMO removal	36
Pericardial tumor resection	34
Pulmonary atresia repair with right ventricular outflow tract reconstruction or pulmonary arterioplasty	31
Unifocalization of the major aortopulmonary collateral arteries	30
Adjustment of systemic-to-pulmonary artery shunt	29
Surgical repair of cor triatriatum	29
Aortopexy	27
Tricuspid atresia surgery (bidirectional Glenn procedure)	27
Division of double aortic arch	24
Implantable loop recorder removal	21
Percutaneous aortic valvuloplasty	21
Total	19,534

*PCPS* Percutaneous cardiopulmonary support, *ECMO* Extracorporeal membrane oxygenation, *ICD* Implantable cardioverter-defibrillator, *VAD* Ventricular assist device

**Table 23:** Great vessels (total; 21,174 cases)

Procedure	Case
Ascending aortic aneurysm replacement	3713
Aortic aneurysm repair with frozen elephant trunk	3190
Abdominal aortic aneurysm replacement (with branch vessel reconstruction)	3125
Abdominal aortic aneurysm replacement (without branch vessel reconstruction)	3085
Total arch replacement	2671
Combined surgery of ascending aortic and aortic arch repair	2199
Thoracoabdominal aortic aneurysm repair	731
Descending aortic aneurysm repair	657
Debranching (1–4 vessels) endovascular aneurysm repair	288
Vascular grafting or bypass for visceral artery	227
Abdominal aortic aneurysm replacement with renal artery clamping	215
Graft replacement for ruptured abdominal aortic aneurysm	203
Graft replacement with renal artery clamp for abdominal aortic aneurysm	202
Vascular grafting or bypass for aorta	191
Aneurysmorrhaphy	127
Vascular grafting or bypass for intrathoracic artery	103
Aortic valvuloplasty or anastomosis	86
Vascular grafting or bypass for the iliac artery	83
Stent grafting for aortic arch branches	78
Total	21,174

**Table 24:** Great veins (total; 407 cases)

Procedure	Case
Venous reconstruction of intra-abdominal vein	129
Venous anastomosis of intra-abdominal vein	126
Venous grafting or bypass surgery for intra-abdominal vein	71
Venous reconstruction of intrathoracic veins	44
Inferior vena cava injury repair with suture hemostasis	37
Total	407

**Table 25:** Other great vessel surgeries (total; 27,461 cases)

Procedure	Case
Stent grafting of the abdominal aorta	13,206
Surgical thrombectomy (artery, without thoracotomy or laparotomy)	6705
Stent grafting of the thoracic aorta	6351
Aortic cross-clamping (thoracotomy)	291
Inferior vena cava filter placement	236
Thrombectomy (with laparotomy)	121
Pulmonary vein reconstruction	110
Stent grafting of the thoracoabdominal aorta	105
Aortic cross-clamping with a balloon catheter	82
Pulmonary thromboendarterectomy	77
Open venous thrombectomy	56
Aortic coarctation surgery with Damus-Kaye-Stansel (DKS) anastomosis	43
Thoracic aortic wrapping	39
Thoracic aortic pseudoaneurysm repair	39
Total	27,461

**Fig. 4:**
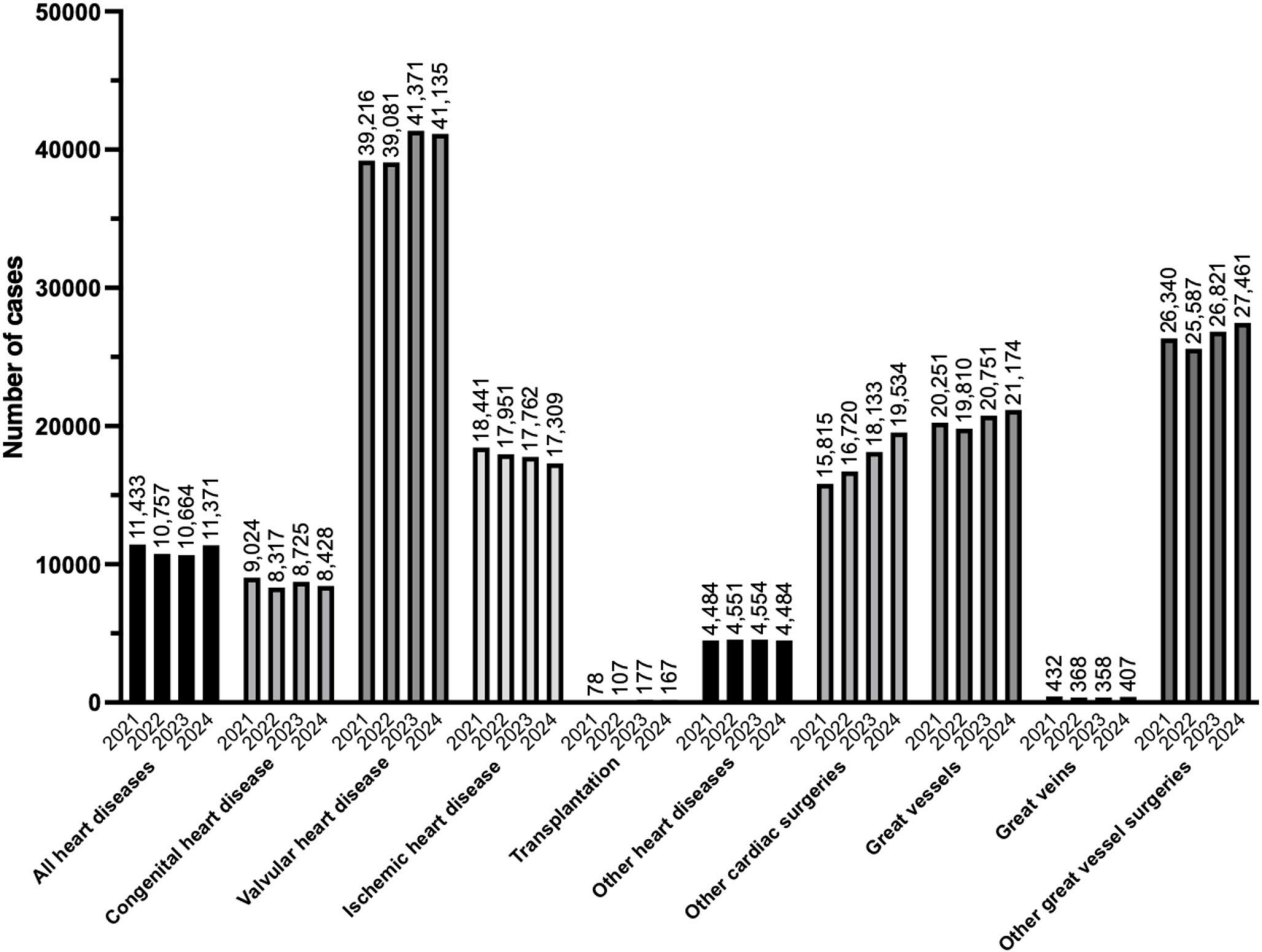
Trends in surgical procedure volumes from 2021 to 2024 in the category of cardiovascular surgery

### Peripheral vascular surgery

In 2024, 223,422 peripheral vascular surgical procedures were registered in the NCD, which was increased in more than 2700 cases compared with 2023. Since 2021, the number of registered cases in this category has consistently increased. The procedure with the highest number in this category was endovascular treatment for the extremity arteries (n = 59,899), followed by endovenous ablation of the lower extremity varicose veins (n = 28,569), and peripheral arteriovenous fistula creation (n = 21,311).

Tables 26, 27, 28, 29 and Fig. 5 show the number of surgeries in each subcategory (arteries, veins, other vascular systems, and other peripheral vascular surgery, respectively). The number of endovascular treatments for the extremity arteries showed the largest year-to-year increase among all procedures, rising by more than 5000 cases from 54,798 in 2023 [3]. Consequently, the “arteries” subcategory demonstrates a pronounced increasing trend over the past four years. In contrast, endovenous ablation of the lower extremity varicose veins decreased by 1584 cases and peripheral arteriovenous fistula creation also decreased by 869 cases compared with the previous year.

**Table 26:** Arteries (total; 111,104 cases)

Procedure	Case
Endovascular treatment for the extremity arteries	59,899
Stent grafting of the abdominal aorta	13,206
Surgical thrombectomy (without thoracotomy or laparotomy)	6705
Stent grafting of the thoracic aorta	6351
Thromboendarterectomy (other arteries)	3358
Abdominal aortic aneurysm replacement (with branch vessel reconstruction)	3125
Abdominal aortic aneurysm replacement (without branch vessel reconstruction)	3085
Arterial reconstruction or anastomosis (other arteries)	2991
Stent grafting of the iliac artery	2288
Arterial reconstruction or anastomosis for the femoropopliteal artery	1383
Vascular grafting or bypass for the tibial and foot arteries	1327
Vascular grafting or bypass for the femoral artery	1247
Vascular grafting or bypass for the below-knee popliteal artery	1050
Percutaneous angioplasty for the limb arteries (with a stent graft)	1027
Debranching TEVAR with 1 or 2 bypass grafts	656
Peripheral artery suture hemostasis or anastomosis	503
Arterial reconstruction or anastomosis for the intra-abdominal arteries (excluding aorta)	409
Percutaneous aspiration thrombectomy of the limb arteries	280
Vascular grafting or bypass for the intra-abdominal arteries	227
Peripheral aneurysm resection (other types)	223
Peripheral aneurysm resection (with anastomosis or grafting)	215
Peripheral aneurysm resection	195
Percutaneous angioplasty for the renal artery	186
Arterial reconstruction or anastomosis for the finger arteries (hand or foot)	164
Fenestrated and branched TEVAR (with bridging-stent or stent grafts including in-situ fenestration)	126
Fenestrated and branched EVAR (with bridging-stent or -stent grafts, including iliac branch endoprosthesis [IBE])	119
Stent grafting for the thoracoabdominal aorta	105
Vascular grafting or bypass for the iliac artery	83
Stent grafting for aortic arch branches	78
Stent grafting for the visceral arteries	74
CHIMPS EVAR	68
Thromboendarterectomy (Aorta)	64
Fenestrated and branched thoracoabdominal EVAR (with bridging stent grafts)	62
Percutaneous stent placement for the cervical and cerebral arteries	56
Carotid endarterectomy (CEA)	42
Renal artery revascularization	39
Iliac artery suture hemostasis or anastomosis	39
CHIMPS TEVAR	28
Aortic arch branch reconstruction	21
Total	111,104

*TEVAR* Thoracic endovascular aortic repair, *EVAR* Endovascular aneurysm repair, *CHIMPS* Chimney, periscope, snorkel

**Table 27:** Veins (total; 36,657 cases)

Procedure	Case
Endovenous ablation of the lower extremity varicose veins	28,569
Excision of the lower extremity varicose veins	3835
Stripping of the lower extremity varicose veins	2497
Venous thrombectomy (non-laparotomy)	732
Percutaneous aspiration thrombectomy of extremity	454
Venous grafting or bypass (other veins)	308
Peripheral venous suture hemostasis or anastomosis	135
Venous grafting or bypass for the intra-abdominal veins	71
Venous thrombectomy (laparotomy)	56
Total	36,657

**Table 28:** Other vascular systems (total; 30,817 cases)

Procedure	Case
Peripheral arteriovenous fistula creation	21,311
Arterial grafting or bypass (other arteries)	4714
Peripheral arteriovenous fistula creation using synthetic graft	1548
Percutaneous angioplasty for an arteriovenous fistula (with stent graft)	1351
Arterial grafting or bypass for the head and neck arteries	552
Peripheral arteriovenous fistula creation with ulnar vein transposition	283
Vessel ligation (laparotomy)	250
Arterial grafting or bypass for the aorta	191
Arterial grafting or bypass for the intrathoracic arteries	103
Vessel ligation (thoracotomy)	90
Aortic repair or anastomosis	86
Lymphatic vessel anastomosis	85
Percutaneous angioplasty for artery injury of an extremity (with stent graft)	70
Percutaneous embolization (retroperitoneum) for emergency hemostasis	44
IVC injury repair with suture hemostasis	37
Venous injury repair or ligation of the intra-abdominal veins (excluding IVC)	37
Lymphaticovenous anastomosis	33
Percutaneous embolization for limb artery injury (emergency hemostasis)	32
Total	30,817

*IVC* Inferior vena cava

**Table 29:** Other peripheral vascular surgeries (total; 44,844 cases)

Procedure	Case
Percutaneous venoplasty for limb veins	6506
Endovascular embolization for lower extremity varicose veins	6325
Arterial embolization	3497
Surgical removal of PCPS/ECMO	3134
Vascular exposure surgery (artery)	2908
Sclerotherapy for lower extremity varicose veins	2863
Vascular embolization (head, thoracic, or abdominal vessels)	2628
Venous graft harvesting	2330
Vessel ligation (non-thoracotomy or non-laparotomy)	2187
Venous graft harvesting (great saphenous vein)	2124
High ligation of lower extremity veins	1782
Internal thoracic artery harvesting	1277
Surgical insertion of PCPS/ECMO	991
Toe amputation	900
Arterial graft harvesting	831
Arteriovenous fistula takedown	595
Transfemoral amputation or above-knee amputation	498
Venoplasty (other veins)	465
Percutaneous cardiopulmonary support (PCPS) insertion	460
Below-knee amputation	318
Foot amputation	252
Surgical removal of intra-aortic balloon pump (IABP)	184
Radial artery harvesting	165
Percutaneous removal of intravascular foreign body	161
Venous anastomosis (other veins)	152
Surgical insertion of intra-aortic balloon pump (IABP)	136
Inferior vena cava (IVC) filter removal	125
Arterial reconstruction or anastomosis (intrathoracic artery, excluding aorta)	122
Portal vein embolization (via ileal vein)	119
Percutaneous embolization (pelvis)	115
Vascular foreign body removal via arteriotomy	107
Percutaneous angioplasty for mesenteric vessels (with stent placement)	81
Arterial flap surgery	74
Iliac artery aneurysm surgery	74
Gastroepiploic artery harvesting	63
Autologous free composite tissue transplantation	63
Free flap transplantation	60
Aortic coarctation surgery with Damus-Kaye-Stansel (DKS) anastomosis	43
Venoplasty and anastomosis for the finger veins (hand or foot)	42
Finger amputation	25
Intra-aortic balloon pumping (every 3 h)	22
Portal vein embolization (transhepatic)	20
Internal iliac artery embolization (open)	20
Total	44,844

*PCPS* Percutaneous cardiopulmonary support, *ECMO* Extracorporeal membrane oxygenation, *IABP* Intra-aortic balloon pump, *IVC* Inferior vena cava

**Fig. 5:**
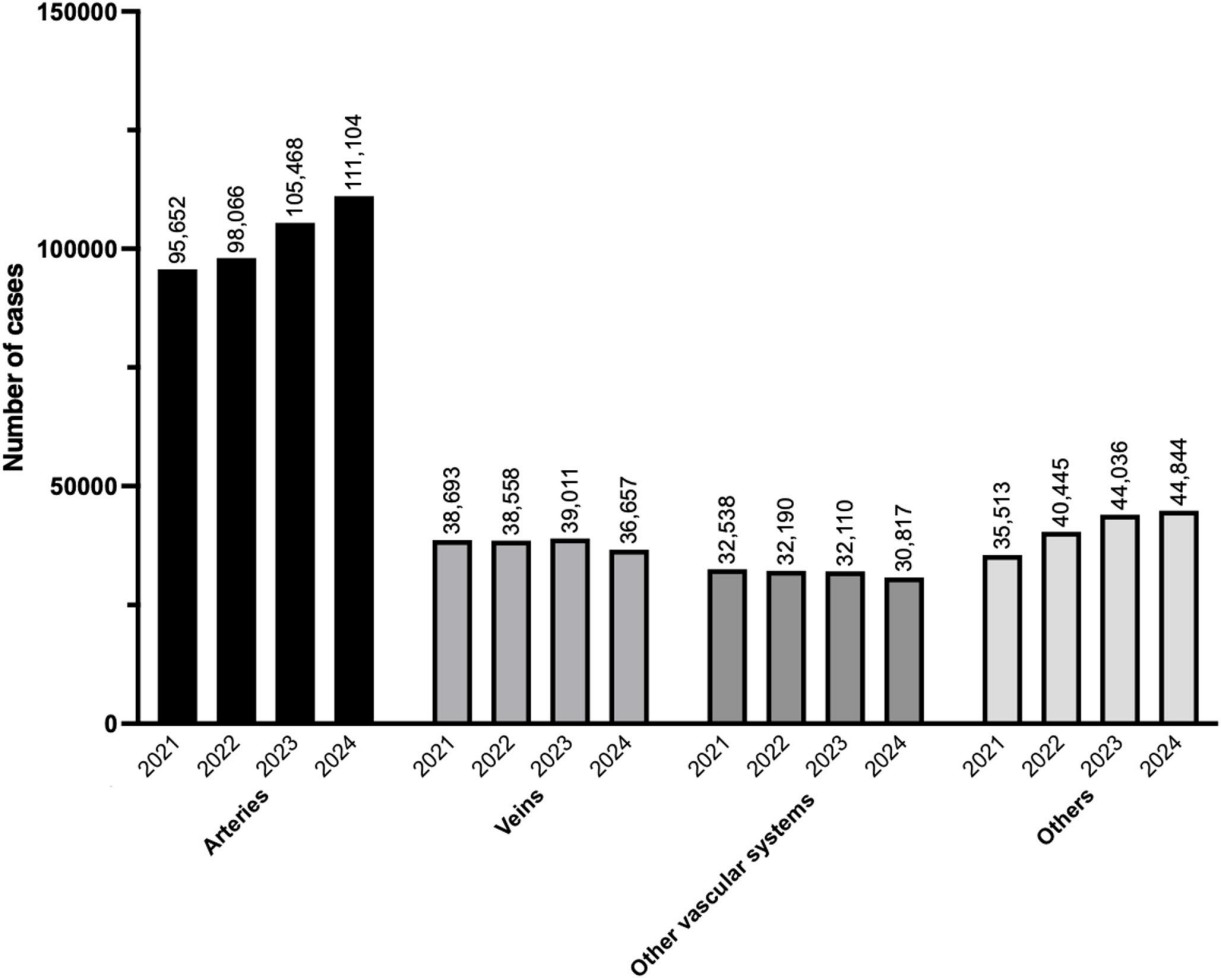
Trends in surgical procedure volumes from 2021 to 2024 in the category of peripheral vascular surgery

### Surgery of the head and neck/body surface/endocrine system

In 2024, 160,540 surgical procedures in the category of the head and neck/body surface/endocrine system were registered in the NCD. Since 2022, the number of registered cases in this category has slightly and consistently increased every year. The procedure with the highest number in this category was wound treatment (≥ 6 years), length < 5 cm, not involving muscle or organ (n = 35,493), followed by skin incision (length < 10 cm) (n = 13,723), and incision and drainage of a perianal abscess (n = 8978). The ranking of these three procedures was identical to that of the previous year, and their registered case numbers showed no substantial changes.

Tables 30, 31, 32, 33 and Fig. 6 show the number of surgeries in each subcategory (skin and soft tissues; neck; thyroid and parathyroid glands; and adrenal and gonadal glands, respectively).

**Table 30:** Skin and soft tissues (total; 122,705 cases)

Procedure	Case
Wound treatment (≥ 6 years), length < 5 cm, not involving muscle or organ	35,493
Skin incision (length < 10 cm)	13,723
Incision and drainage of a perianal abscess	8978
Wound treatment (≥ 6 years, length 5–10 cm, not involving muscle or organ)	7142
Wound treatment (≥ 6 years, length < 5 cm, involving muscle or organ)	6337
Lymph node excision (diameter < 3 cm)	5434
Excision of a subcutaneous benign tumor (non-exposed area, diameter < 3 cm)	3271
Wound treatment (≥ 6 years, length 5–10 cm, involving muscle or organ)	3263
Excision of a benign skin tumor (non-exposed area, diameter < 3 cm)	2912
Lymph node excision (diameter ≥ 3 cm)	2781
Wound treatment (≥ 6 years, length ≥ 10 cm, involving muscle or organ)	2569
Excision of a subcutaneous benign tumor (non-exposed area, diameter 3–6 cm)	2369
Excision of a benign skin tumor (exposed area, diameter < 2 cm)	2295
Simple ingrown nail surgery	1853
Wound treatment (< 6 years, length < 2.5 cm, not involving muscle or organ)	1797
Wound treatment (≥ 6 years, length ≥ 10 cm, not involving muscle or an organ)	1751
Excision of a benign skin tumor (non-exposed area, diameter 3–6 cm)	1650
Excision of a subcutaneous benign tumor (exposed area, diameter < 2 cm)	1482
Excision of a subcutaneous benign tumor (exposed area, diameter 2–4 cm)	1298
Incision and drainage of perirectal abscess	1251
Excision of a subcutaneous foreign body	1143
Excision of a benign skin tumor (exposed area, diameter 2–4 cm)	1089
Excision of a subcutaneous benign tumor (non-exposed area, diameter ≥ 6 cm)	909
Nail plate removal	909
Simple excision of a malignant skin tumor	870
Wound treatment (< 6 years, length 2.5–5 cm, not involving muscle or organ)	583
Wound treatment (< 6 years, length 2.5–5 cm, involving muscle or organ)	558
Skin incision (length 10–20 cm)	531
Ingrown nail surgery with nail bed/matrix involvement	525
Excision of a subcutaneous benign tumor (exposed area, diameter ≥ 4 cm)	497
Incision and drainage for felon (soft tissue infection)	493
Excision of a benign skin tumor (non-exposed area, diameter ≥ 6 cm)	439
Abdominal wall tumor excision (without reconstructive surgery)	423
Excision of a benign skin tumor (exposed area, diameter ≥ 4 cm)	417
Lymph node biopsy (needle puncture)	389
Excision of a subcutaneous hematoma	378
Wound treatment (< 6 years, length 5–10 cm, involving muscle or organ)	370
Wound treatment (< 6 years, length < 2.5 cm, involving muscle or organ)	360
Split-thickness skin graft (≥ 200 cm^2^)	339
Excision of fa oreign body from muscle	304
Surgery for axillary osmidrosis (flap method)	259
Incision and drainage of abdominal wall abscess	244
Wound treatment (< 6 years, length ≥ 10 cm, involving muscle or organ)	224
Flap creation, transfer, resection, or delayed flap procedure (non-exposed, non-mucosal, non-joint area, < 25 cm^2^)	161
Skin biopsy	156
Split-thickness skin graft (100–200 cm^2^)	144
Incisional biopsy of subcutaneous soft tissue tumor (trunk)	120
Skin incision (length ≥ 20 cm)	120
Skin shaving (< 25 cm^2^)	111
Full-thickness skin graft (< 25 cm^2^)	111
Full-thickness skin graft (25–100 cm^2^)	103
Split-thickness skin graft (25–100 cm^2^)	102
Excision of a benign soft tissue tumor (trunk)	98
Open liver cyst fenestration	87
Scar contracture release	82
Excision of a breast foreign body	80
Flap creation, transfer, resection, or delayed flap procedure (exposed area, < 25 cm^2^)	76
Arterial flap surgery	74
Incision and drainage of chest wall abscess	72
Foreign body removal from palm	65
Wound treatment (< 6 years, length 5–10 cm, not involving muscle or organ)	65
Autologous free composite tissue transplantation	63
Free flap transplantation	60
Stump revision (soft tissue only, toe)	57
Pedicled musculocutaneous flap transfer	55
Full-thickness skin graft (100–200 cm^2^)	51
Incision and drainage of deep cervical abscess	48
Flap creation, transfer, resection, or delayed flap procedure (non-exposed, non-mucosal, non-joint area, 25–100 cm^2^)	42
Lymph node abscess incision and drainage	41
Flap creation, transfer, resection, or delayed flap procedure (non-exposed, non-mucosal, non-joint area, ≥ 100 cm^2^)	40
Foreign body removal from sole	39
Split-thickness skin graft (< 25 cm^2^)	39
Wound treatment (age under 6, length ≥ 10 cm, not involving muscle or organ)	36
Simple resection of a malignant neck tumor	34
Full-thickness skin graft (≥ 200 cm^2^)	33
Flap creation, transfer, resection, or delayed flap procedure (joint area, < 25 cm^2^)	32
Excision of a superficial hemangioma (exposed area other than face or head, diameter < 3 cm)	30
Flap creation, transfer, resection, or delayed flap procedure (exposed area, 25–100 cm^2^)	30
Excision of a superficial hemangioma (non-exposed area, diameter 3–6 cm)	28
Flap creation, transfer, resection, or delayed flap procedure (exposed area ≥ 100 cm^2^)	27
Wide excision of a malignant skin tumor	26
Mediastinal lymph node dissection (parasternal)	26
Excision of a superficial hemangioma (non-exposed area, diameter < 3 cm)	25
Allogeneic skin grafting using cultured skin grafts	25
Pedicled muscle flap transfer	24
Incisional biopsy of a soft tissue tumor (trunk)	23
Esophageal foreign body removal (cervical approach)	22
Stump revision (soft tissue only, finger)	20
Total	122,705

**Table 31:** Neck (total; 18,817 cases)

Procedure	Case
Tracheostomy	7486
Thyroidectomy (without lateral neck dissection)	3237
Axillary lymph node dissection	3094
Total or subtotal thyroidectomy (without lateral neck dissection)	1625
Total or subtotal thyroidectomy (with unilateral lateral neck dissection)	823
Thyroidectomy (with lateral neck dissection)	505
Cervical lymph node dissection	463
Unilateral neck dissection	326
Total or subtotal thyroidectomy (with bilateral lateral neck dissection)	233
Endoscopic thyroid resection for malignant thyroid tumor	207
Retroperitoneal lymph node dissection	192
Inguinal and femoral lymph node dissection	188
Thyroglossal duct cyst excision	164
Bilateral neck dissection	127
Excision of branchial fistula or branchial cyst	80
Endoscopic total or subtotal thyroidectomy for malignant thyroid tumor	39
Parotid tumor excision	28
Total	18,817

**Table 32:** Thyroid and parathyroid glands (total; 9,905 cases)

Procedure	Case
Partial thyroidectomy (thyroid nodule excision, unilateral)	4138
Parathyroidectomy	1850
Total thyroidectomy for Graves’ hyperthyroidism	1627
Partial thyroidectomy (thyroid nodule excision, bilateral)	751
Endoscopic hemithyroidectomy	438
Subtotal thyroidectomy for Graves’ hyperthyroidism	274
Completion total thyroidectomy	208
Fasciotomy or fascial incision	125
Parathyroidectomy with autotransplantation	116
Endoscopic parathyroid tumor resection	81
Thyroid tissue biopsy via excisional biopsy	74
Thyroid biopsy via open incision	65
Endoscopic-assisted thyroidectomy	56
Endoscopic total thyroidectomy for Graves’ disease	55
Laryngopharyngeal surgery for malignant tumor (with cervical, thoracic, or abdominal reconstruction)	26
Extensive parathyroid cancer resection	21
Total	9905

**Table 33:** Adrenal and gonadal glands (total; 9,113 cases)

Procedure	Case
Orchiopexy for undescended testis (extra-abdominal)	4379
Circumcision (circular excision)	619
Surgical repair of hydrocele of the canal of Nuck	503
Repair of communicating hydrocele	460
Resection of a retroperitoneal malignant tumor	410
Laparoscopic resection of a retroperitoneal tumor	276
Orchiectomy	272
Extensive resection of a retroperitoneal malignant tumor	255
Surgery for testicular torsion (with contralateral orchiopexy)	197
Ovarian tumor excision (open surgery)	185
Ovarian tumor excision (laparoscopic)	168
Laparoscopic orchiopexy for intra-abdominal undescended testis	155
Laparoscopic adrenal tumor resection	127
Adrenalectomy	121
Circumcision (dorsal slit method)	116
Surgery for testicular torsion (without contralateral orchiopexy)	112
Orchiopexy for undescended testis (intra-abdominal)	108
Surgery for testicular torsion	91
Laparoscopic partial oophorectomy	85
Open partial oophorectomy	84
Laparoscopic retroperitoneal tumor biopsy	61
Laparoscopic orchiectomy for undescended testis	54
Open adrenal tumor resection	48
Adrenal tumor resection (medullary tumor, pheochromocytoma)	47
Orchiopexy for contralateral testis during testicular torsion	42
Orchiectomy for undescended testis	41
Laparoscopic resection of an adrenal malignant tumor	39
Simple adrenal malignant tumor resection (including pheochromocytoma)	30
Circumcision (frenuloplasty or phimosis repair)	28
Total	9113

**Fig. 6:**
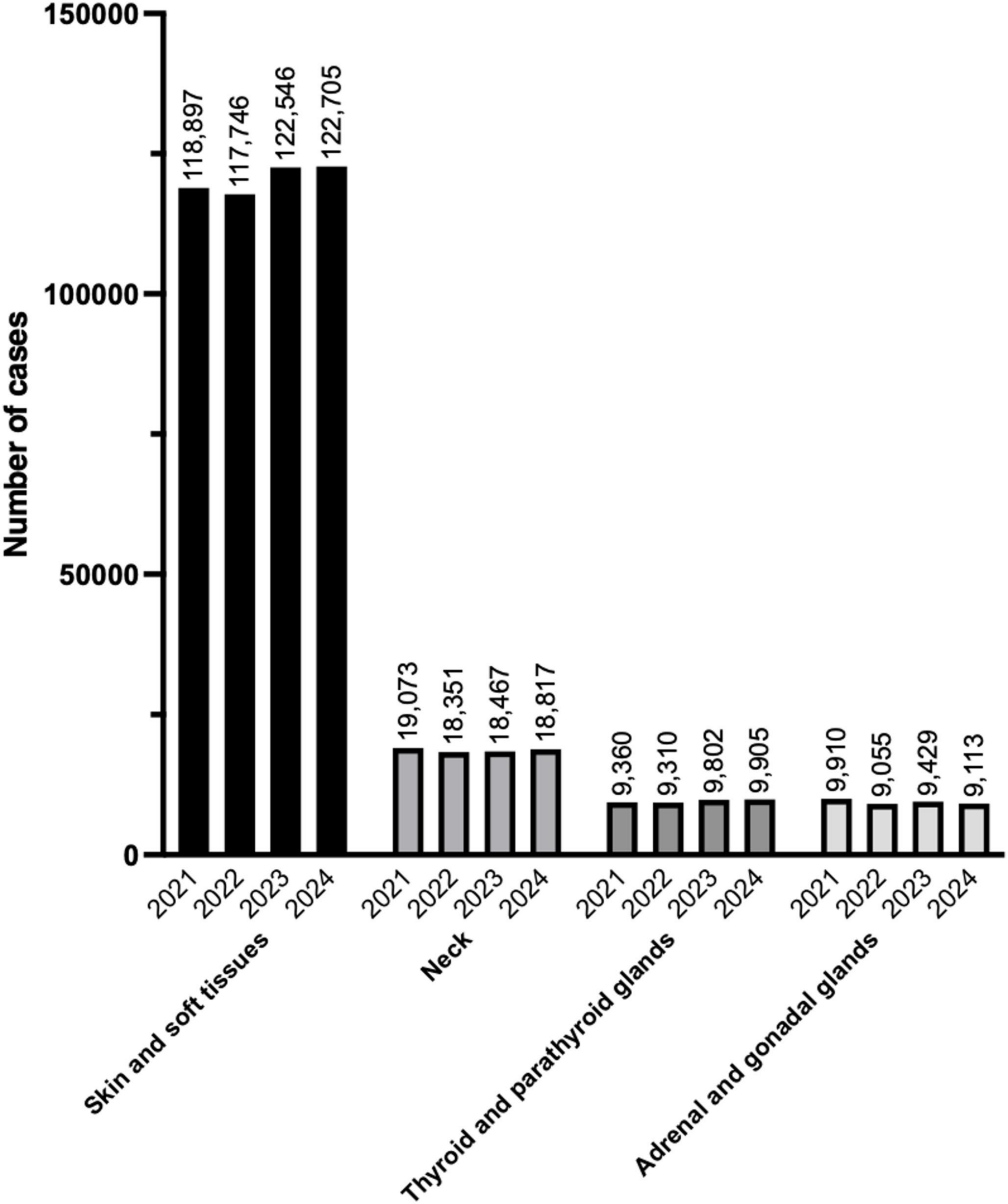
Trends in surgical procedure volumes from 2021 to 2024 in the category of surgery of the head and neck/body surface/endocrine system

### Pediatric surgery

In 2024, 42,660 pediatric surgical procedures were registered in the NCD. The procedure with the highest number in this category was laparoscopic inguinal hernia surgery (n = 8948), followed by open inguinal hernia surgery (n = 5080), and orchiopexy for undescended testis (extra-abdominal) (n = 4359). Although the ranking of these three procedures was unchanged from the previous year, laparoscopic inguinal hernia surgery has shown a consistent upward trend over the past three years, whereas open inguinal hernia surgery has exhibited a steady decline.

Tables 34, 35, 36, 37, 38, 39, 40, 41 and Fig. 7 show the number of surgeries in each subcategory (body surface; thorax; diaphragm; gastroenterological system; nutritional management; tumor; transplantation; and other pediatric surgical procedures, respectively).

**Table 34:** Body surface (total; 24,439 cases)

Procedure	Case
Laparoscopic inguinal hernia surgery	8948
Open inguinal hernia surgery	5080
Orchiopexy for undescended testis (extra-abdominal)	4359
Umbilical hernia surgery (rectus abdominis diastasis)	3915
Circumcision (circular excision)	546
Laparoscopic orchiopexy for intra-abdominal testis	155
Excision of a benign skin tumor (exposed area, diameter < 2 cm)	151
Excision of a subcutaneous benign tumor (exposed area, diameter < 2 cm)	144
Surgery for hydrocele of the canal of Nuck	129
Excision of a benign skin tumor (non-exposed area, diameter < 3 cm)	110
Circumcision (dorsal slit)	108
Orchiopexy for intra-abdominal testis	106
Abdominal wall reconstruction (abdominal wall rupture or omphalocele)	96
Excision of a subcutaneous benign tumor (non-exposed area, diameter < 3 cm)	95
Excision of a subcutaneous benign tumor (exposed area, diameter 2–4 cm)	75
Open abdominal wall incisional hernia surgery	64
Excision of branchial fistula or cyst	64
Excision of a benign skin tumor (exposed area, diameter 2–4 cm)	61
Laparoscopic orchiectomy for undescended testis	49
Excision of a subcutaneous benign tumor (non-exposed area, diameter 3–6 cm)	43
First-stage omphalocele repair	40
Open orchiectomy for undescended testis	30
Excision of a benign skin tumor (non-exposed area, diameter 3–6 cm)	25
Excision of lymphangioma (diameter ≥ 3 cm)	24
Piriformis fossa fistula/cyst excision	22
Total	24,439

**Table 35:** Thorax (total; 758 cases)

Procedure	Case
Endoscopic pectus excavatum repair	139
Pectus bar removal	111
Pectus excavatum repair (sternal elevation)	100
Thoracoscopic lobectomy	76
Exploratory thoracotomy	72
Thoracoscopic mediastinal tumor resection	58
Open lobectomy	56
Thoracoscopic wedge resection (two or more sites) for pulmonary cyst	52
Thoracoscopic debridement for empyema	45
Thoracoscopic lung plication	27
Open mediastinal tumor resection	22
Total	758

**Table 36:** Diaphragm (total; 144 cases)

Procedure	Case
Congenital diaphragmatic hernia surgery (transabdominal, direct suture)	57
Congenital diaphragmatic hernia surgery (transabdominal, with synthetic patch)	44
Thoracoscopic surgery for congenital diaphragmatic hernia (direct suture)	43
Total	144

**Table 37:** Gastroenterological system (total; 11,378 cases)

Procedure	Case
Laparoscopic simple appendectomy	4041
Laparoscopic complex appendectomy	1223
Laparoscopic-assisted gastrostomy (including percutaneous endoscopic, percutaneous, and open approaches)	570
Stoma creation	486
Stoma closure with bowel resection	391
Exploratory laparotomy for diagnosis and biopsy	293
Open gastrostomy	272
Open small bowel resection	252
Pyloromyotomy for hypertrophic pyloric stenosis	245
Incision and drainage of perianal abscess	241
Open appendectomy	221
Acute diffuse peritonitis surgery with drainage of intraperitoneal abscess	212
Low perineal anorectal reconstruction for imperforate anus	207
Open surgery for intestinal adhesions	206
Surgery for intestinal malrotation	178
Enterostomy creation	172
Radical surgery for complex fistula-in-ano	168
Endoscopic gastrostomy creation	132
Laparoscopic Hirschsprung disease surgery	116
Definitive surgery for esophageal atresia (Gross type C)	103
Laparoscopic surgery for intestinal adhesions	97
Meckel’s diverticulectomy	92
Surgery for duodenal atresia or stenosis (diamond anastomosis)	90
Enterostomy closure with bowel resection	83
Surgery for biliary atresia	80
Surgery for congenital intestinal atresia (with bowel resection)	77
Laparoscopic small bowel resection	71
Placement of abdominal drain	69
Open limited colectomy	66
Disinvagination (invasive) (including Hutchinson’s maneuver)	62
Laparoscopic surgery for choledochal cyst	61
Surgery for choledochal cyst	59
Localized intraperitoneal abscess surgery (appendiceal abscess)	54
Laparoscopic splenectomy	54
Laparoscopic high anorectal reconstruction for imperforate anus	52
Laparoscopic disinvagination (including Hutchinson’s maneuver)	50
Laparoscopic hiatal hernia surgery	49
Open pyloroplasty	49
Laparoscopic pyloromyotomy for hypertrophic pyloric stenosis	46
Laparoscopic limited colectomy	41
Hirschsprung disease surgery	36
Anal polyp resection	36
Thoracoscopic esophageal atresia repair	34
Surgery for congenital intestinal atresia (without bowel resection)	33
Laparoscopic surgery for intestinal malrotation	30
Stoma closure without bowel resection	30
Intermediate anorectal reconstruction for imperforate anus (PSARP)	28
Esophageal variceal ligation	27
Intermediate anorectal reconstruction for imperforate anus (sacroperineal approach)	26
Surgery for intestinal duplication	24
Colostomy creation	22
Open gastric perforation repair	21
Total	11,378

**Table 38:** Nutritional management (total; 2102 cases)

Procedure	Case
Placement of central venous nutrition port (head, neck, or other sites)	1750
Central venous nutrition catheter placement (venotomy)	262
Placement of central venous nutrition port (limbs)	90
Total	2102

**Table 39:** Tumors (total; 445 cases)

Procedure	Case
Ovarian tumor excision (open surgery)	72
Ovarian tumor excision (laparoscopic surgery)	58
Adnexal tumor excision (open surgery)	55
Excision of omental, mesenteric, and retroperitoneal tumors (without bowel resection)	45
Laparoscopic partial oophorectomy	39
Simple nephrectomy for renal malignancy	32
Uterine Adnexal tumor excision (laparoscopic surgery)	31
Uterine adnexal biopsy (excisional)	26
Lung resection (wedge resection)	23
Sacrococcygeal teratoma surgery (superficial)	22
Partial liver resection (others)	22
Simple orchiectomy for testicular malignancy (including high inguinal orchiectomy)	20
Total	445

**Table 40:** Transplantation (total; 166 cases)

Procedure	Case
Living donor partial liver transplantation	106
Allogeneic kidney transplantation	34
Back table surgery for liver transplantation (living donor)	26
Total	166

**Table 41:** Other pediatric surgeries (total; 3,228 cases)

Procedure	Case
Lingual frenuloplasty	548
Esophageal dilation (balloon catheter)	434
Hypospadias repair	297
Vesicoureteral reflux surgery (collagen and hyaluronic acid injection)	232
Excision of urachal remnant	173
Ureteral stent removal	167
Vesicoureteral reflux surgery	165
Peritoneal dialysis catheter placement	114
Ureteral stent placement	108
Excision of accessory auricle	96
Sclerotherapy for superficial lymphangioma	91
Labial frenuloplasty	88
Excision of congenital auricular sinus	85
Ureteral reimplantation with anti-reflux mechanism	75
Laryngotracheal separation	72
Surgery for buried penis	67
Sclerotherapy for deep lymphangioma	65
Pyeloplasty (including ureteropelvic junction repair)	64
Laparoscopic pyeloplasty (including ureteropelvic junction repair)	56
Labial adhesion repair (excluding simple adhesion release)	49
Cystostomy creation	36
Ureteral stent exchange	33
Bronchial foreign body removal (bronchoscopic)	28
Thoracoscopic diaphragm plication	23
Thoracic diaphragm suture (without patch reconstruction)	21
Endoscopic gastrointestinal foreign body removal (other sites, distal to duodenum)	21
Surgery for laryngeal stenosis (T-tube insertion)	20
Total	3228

**Fig. 7:**
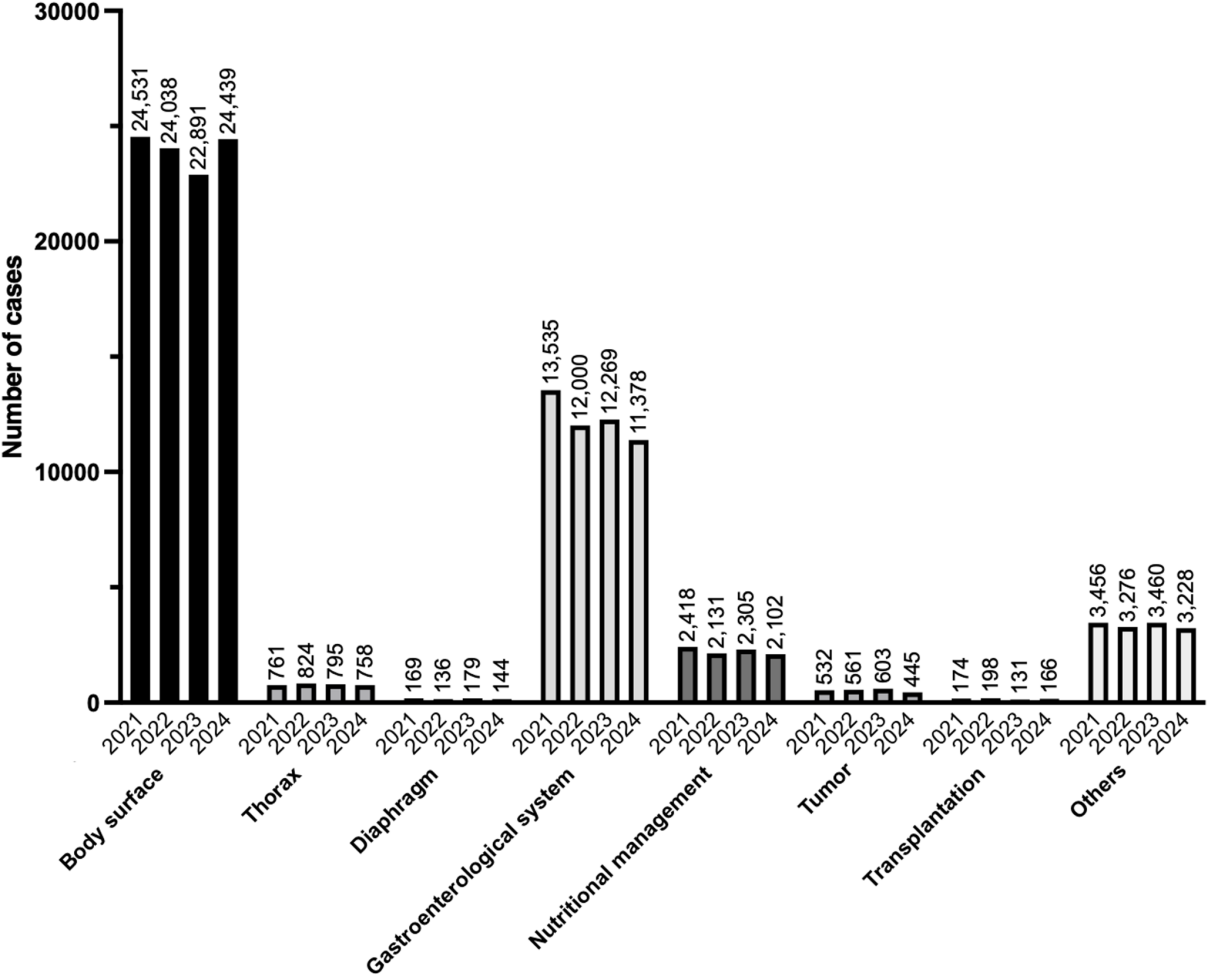
Trends in surgical procedure volumes from 2021 to 2024 in the category of pediatric surgery
